# Diverse and potentially manipulative signalling with ascarosides in the model nematode *C. elegans*

**DOI:** 10.1186/1471-2148-14-46

**Published:** 2014-03-11

**Authors:** Sylvia Anaid Diaz, Vincent Brunet, Guy C Lloyd-Jones, William Spinner, Barney Wharam, Mark Viney

**Affiliations:** 1 School of Biological Sciences, University of Bristol, Woodland Road, Bristol BS8 1UG, UK; 2 School of Chemistry, University of Bristol, Cantock’s Close, Bristol BS8 1TS, UK; 3 Present Address: School of Veterinary Medicine, University of Cambridge, Madingley Road, Cambridge CB3 0ES, UK

**Keywords:** *C. elegans*, Dauer, Arrest, Ascaroside, Signalling

## Abstract

**Background:**

Animals use environmental information to make developmental decisions to maximise their fitness. The nematode *Caenorhabditis elegans* measures its environment to decide between arresting development as dauer larvae or continuing to grow and reproduce. Worms are thought to use ascarosides as signals of population density and this signalling is thought to be a species-wide honest signal. We compared recently wild *C. elegans* lines’ dauer larva arrest when presented with the same ascaroside signals and in different food environments.

**Results:**

We find that the hitherto canonical dauer larva response does not hold among these lines. Ascaroside molecules can, depending on the food environment, both promote and repress dauer larva formation. Further, these recently wild *C. elegans* lines also produce ascaroside mixtures that induce a wide diversity of dauer larva formation responses. We further find that the lines differ in the quantity and ratios of ascaroside molecules that they release. Some of the dauer larva formation responses are consistent with dishonest signalling.

**Conclusions:**

Together, the results suggest that the idea that dauer larva formation is an honestly-signalled *C. elegans*-wide effect does not hold. Rather, the results suggest that ascaroside-based signalling is a public broadcast information system, but where the correct interpretation of that information depends on the worms’ context, and is a system open to dishonest signalling.

## Background

All organisms use information from their environment, which can include con- and hetero-specifics, to make decisions. The model nematode system, *Caenorhabditis elegans,* as other nematodes, makes a developmental decision between developing into a developmentally arrested dauer larva or developing into a growing, non-dauer larval stage [1-4]. Dauer larvae are commonly found in nature, showing the importance of this life-cycle stage in surviving periods when food is not available [5,6]. This developmental decision is environmentally dependent. Dauer larvae are typically formed in environments that are not suitable for continued growth, specifically a low concentration of food and a high conspecific population density [2,7]. Conspecific population density is thought to be signalled by a mixture of ascaroside molecules that worms release into the environment [8-10]. In this scenario for the wild-type strain, the concentration of these molecules are an accurate and honest measure of conspecific population density which can be understood by all members of the species

At least five ascaroside molecules (ascr#1, #2, #3, #4, #8) induce dauer larva formation with ascr#2 being most potent, but acting synergistically with ascr#3 [8,10-13]. The ascaroside ascr#2 is also the most highly produced ascaroside, especially under dauer larva-inducing conditions [14]. *C. elegans* is now known to produce almost 150 different ascarosides and related molecules [15]. This large number of molecules that interact in signalling have the potential to convey complex information beyond, for example, a simple honest signal of conspecific population density.

Previous work in *C. elegans* has considered how signalling for dauer arrest may operate among different genotypes of the species [16]. This work, which was conducted before the discovery of the role of ascaroside molecules in *C. elegans* biology, found that not all genotypes responded similarly to dauer larvae-inducing signals. These observations, with the discovery of the large number of ascaroside molecules, and together with general considerations of animal signalling more widely [17], raised the possibility that ascaroside signalling may evolve to vary among individuals of a species such that, for example, it is not a simple, honest species-wide signal.

To investigate this we characterised the extensive diversity of dauer larva arrest phenotypes among recently isolated *C. elegans* genotypes in response to synthetic ascaroside molecules, and to natural mixtures produced by worms, and characterised the relevant ascaroside production profiles of different genotypes. We find a very substantial diversity of ascaroside signalling, with different lines producing different signals, and lines differing in how they respond to the same signal. We also find that the ascaroside molecules can promote dauer larvae formation in some lines and represses it in others, thus showing that they are not universal promoters of dauer larva formation. We also find that ascaroside signals may be dishonest signals because we find situations where some lines induces others, but not themselves, to form dauer larvae. Together these findings are consistent with the idea that while ascaroside signalling is publically broadcast, correct interpretation of that signal may be semi-private among ecological co-inhabitants or relatives, or may in some other way depend on some other aspect of the worms’ context.

## Results and discussion

### (a) *C. elegans* lines differ in dauer larvae formation in response to synthetic ascarosides and food

We investigated the dauer larvae formation phenotypes of isogenic lines derived from 20 recently wild *C. elegans* isolates (as well as of the standard wild-type, N2) when exposed to chemically synthesised ascr#2 or ascr#3 separately, or as a mixture, combined with two food concentrations, giving a total of six different environments.

**Figure 1:**
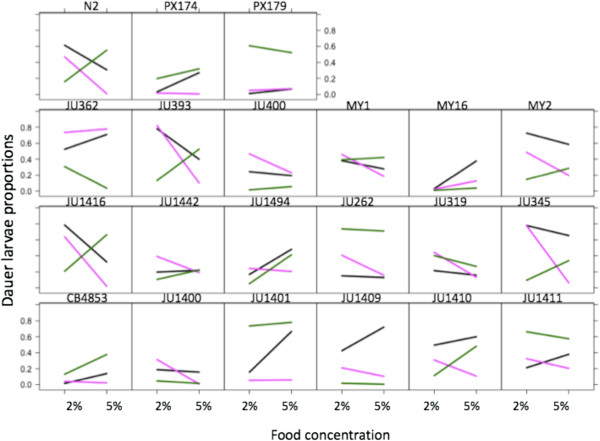
**Significant variation in dauer larvae formation with synthetic ascarosides.** The proportion of dauer larvae formed for each line in the presence of ascr#2 (pink) and ascr#3 (green) or the ascr#2 + ascr#3 mixture (black) at different food conditions (2 and 5%). Values in each condition are shown without error bars for clarity, but the SE are shown in Additional file 1.

There were significant differences among the *C. elegans* lines in how these synthetic ascaroside treatments and food concentrations affected the dauer larva formation of the lines (Line x Synthetic x Food, d.f. = 40, χ^2^ = 877.98, p < 0.001) (Figure 1, Additional file 1). Thus, in the same environmental conditions, the lines’ dauer larva formation responses differed. These among-line differences across these six environments were seen both as different elevations and different slopes (Figure 1). By way of example, ascr#2 induced very little dauer larva formation in line PX174 at either food concentration (*i.e.* low elevation, low slope), but very high dauer formation in JU362 at either food concentration (*i.e.* high elevation, low slope), whereas in JU393 ascr#2 induced high dauer formation at the low food concentration, but very low dauer formation at the high food concentration (*i.e.* high slope).

The hitherto canonical view is that dauer larva formation is induced in low food conditions. However, we find that ascr#3 can reverse this effect and so induce dauer larva formation in high food conditions. Thus, for the N2 wild-type exposed to ascr#3 dauer larva formation increases as food concentration increases. This effect is common (*e.g.* in lines JU393, JU1416, JU1494 and JU1410), though not universal, among the other 20 lines too. Similarly, the ascaroside mixture ascr#2 + ascr#3 can induce comparatively greater dauer larva formation at higher food concentrations, for example in lines MY16, JU1494, JU1401 and JU1409. However, such effects are not seen with ascr#2.

**Figure 2:**
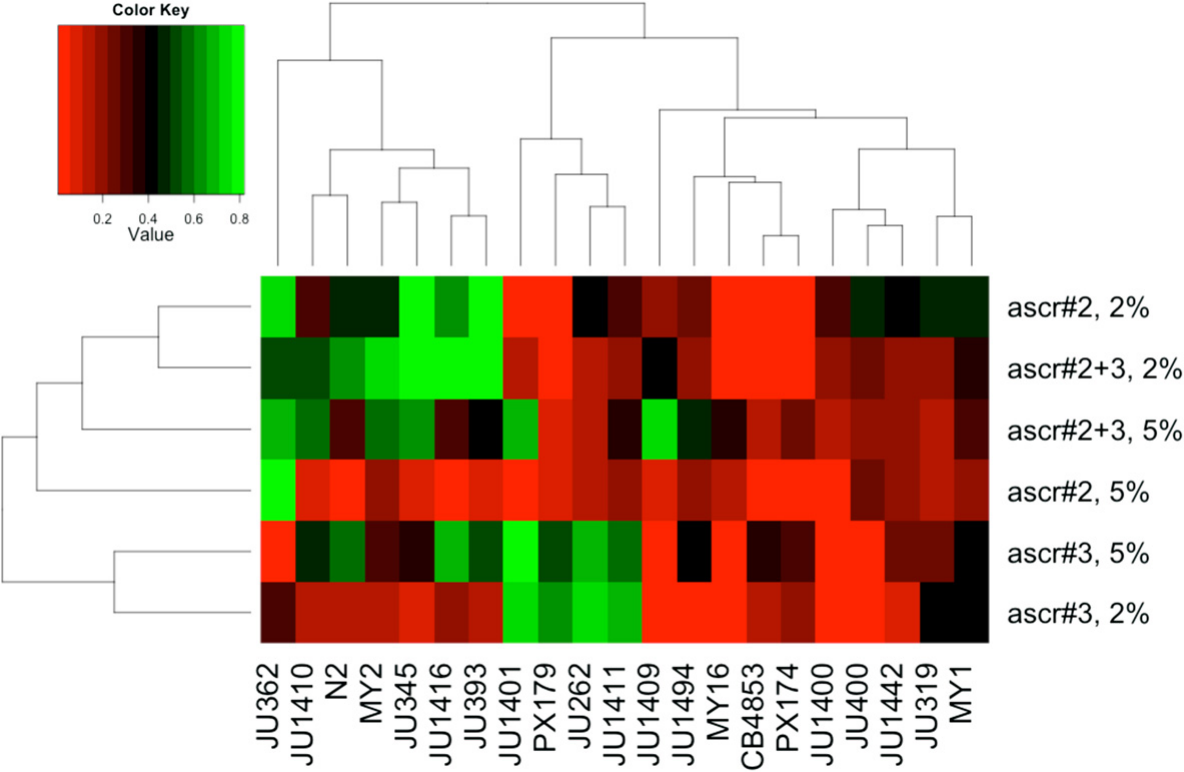
**A mixture of ascr#2 + ascr#3 diversifies dauer larva formation phenotypes among the lines.** A heat map representation of the lines’ dauer larva responses to synthetic ascarosides and food environment, with the lines’ dauer larva formation colour coded, and the values subjected to hierarchical clustering. The top, horizontal, dendrogram shows the relationships of similarities in dauer larva formation among the lines; the side, vertical, dendrogram shows the relationships of similarities in dauer larva formation among the six environmental conditions.

A heat map comparison of the six environmental conditions shows that the mixtures of ascr#2 + ascr#3 generate dauer larva formation phenotypes that are not simple additions of the responses to ascr#2 and ascr#3 when present individually (Figure 2). Such ascaroside interaction effects can therefore generate phenotypic differences among *C. elegans* lines. If the ascaroside molecules act as a combinatorial system, then the very large number of ascaroside molecules acting in combination is a signalling system of potentially huge complexity and sophistication. For the canonical N2 line our results are consistent with previous reports of synergistic effects between these molecules [9,10], such that a greater proportion of dauer larvae developed in the ascaroside mixture compared to each ascaroside separately (0.61 ± 0.02, 0.47 ± 0.03, 0.16 ± 0.08, mean ± SE for ascr#2 + ascr#3, ascr#2 or ascr#3, respectively; Additional file 1). However, this pattern only existed in four other lines (JU1409, JU1410, JU1416, MY2 in the 2% food condition), and thus is not a species-wide phenomenon.

### (b) The natural pheromone mixtures of *C. elegans* lines differ

To investigate the dauer larva formation phenotypes that different *C. elegans* lines could induce, we collected the supernatant from 5 lines (PX174, JU1409, JU1410, MY1 and N2) and then tested their dauer larva induction effects among all lines.

**Figure 3:**
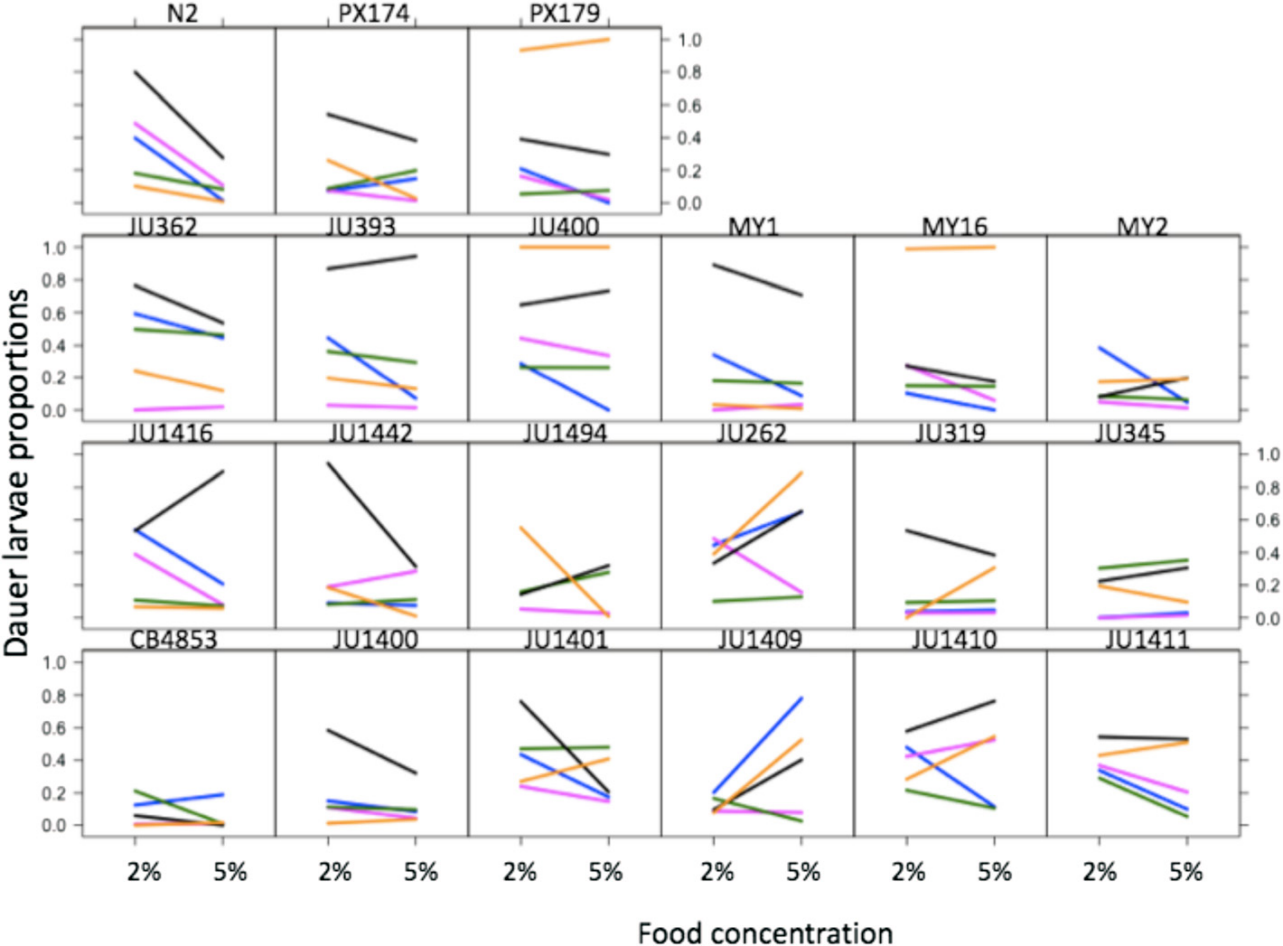
**Significant variation in dauer larvae formation when exposed to natural pheromone mixtures.** The proportion of dauer larvae formed for each line in the presence of pheromone mixtures obtained from JU1409 (blue), JU1410 (pink), MY1 (green), PX174 (yellow) and N2 (black) at different food conditions (2 and 5%). Values in each condition are shown without error bars for clarity, but the SE are shown in Additional file 1. Data for line JU1494 tested with pheromone from JU1409 are not available.

There were significant differences among the *C. elegans* lines in how these natural pheromone mixtures and food concentrations affected the dauer larva formation of the lines (Line x Natural x Food, d.f. = 80, χ^2^ = 1293.8, p < 0.001) (Figure 3, Additional file 1). Among all lines the N2-derived supernatant induced the greatest dauer larva formation at both food concentrations (0.50 ± 0.06 and 0.45 ± 0.05 mean ± SD at 2 and 5% food concentrations, respectively; Additional file 1), while the JU1410-derived supernatant had the lowest induction of dauer larvae (0.19 ± 0.03 and 0.11 ± 0.03 mean ± SD at 2 and 5% food concentrations, respectively; Additional file 1). The lines’ dauer larva formation response to the synthetic ascarosides ascr#2 and ascr#3 did not predict their response to these natural pheromone mixtures, confirming the importance of other molecules in determining dauer larva formation responses.

Comparing these *C. elegans* recently wild lines, the lines were not consistently most sensitive or least sensitive to their own supernatant; N2 and JU1409 were among the most responsive to their own supernatant; lines PX174, JU1410 and MY1 were moderately responsive to their own supernatant (Figure 3). In another free-living nematode, *Pristionchus pacificus*, culture supernatants have been found to preferentially affect dauer arrest in non-self strains [18], but this pattern is not seen in *C. elegans*.

In the presence of these natural pheromone mixtures dauer larva formation can be greatest in a low food environment, (*e.g.* line N2), or greatest in a high food environments (*e.g.* line JU1409) (Figure 3). This latter example shows that dauer larva formation in conditions of low food availability is not a canonical response. Furthermore, the pheromone mixture produced by a line could induce very different dauer larva responses in other lines. For example, the PX174-derived pheromone mixture induced a low-level dauer formation in itself, but almost 100% dauer larva formation in PX179, JU400 and MY16. If these effects also occur in nature, then this could be a strategy by which PX174 induces other strains to form dauer larva, while it itself does not, therefore leaving any food resource for itself. Analogously, the effect of JU1409-derived pheromone on itself, differs substantially from its effect on many other lines (*e.g.* JU393, JU1410). Such a signalling strategy is therefore manipulative, and possibly a dishonest signalling strategy. Beyond this possibility, ascaroside molecules also have other intraspecific roles, such as in mating. There has also been speculation about inter-specific effects of ascarosides [19]. Therefore when observing and interpreting the effects that ascaroside molecules have on dauer larva formation, this does need to be considered with the context that these same molecules may be playing additional roles too.

**Figure 4:**
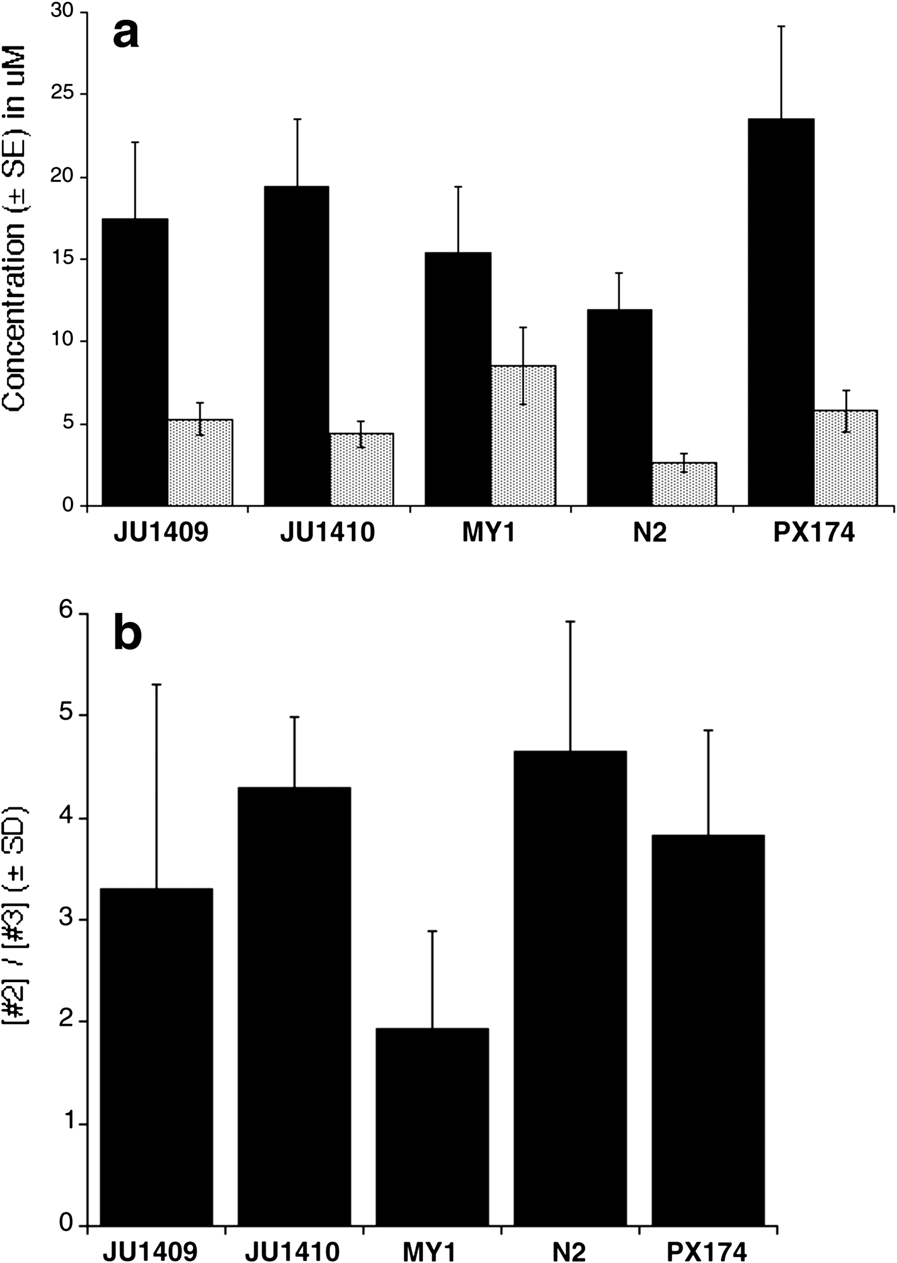
**Variation in ascaroside production among *C. elegans *lines.** The **(a)** mean (± 1 SE) concentration (μM) of ascr#2 (black) and ascr#3 (hatched) produced *per* worm across replicates, **(b)** the (mean ± 1 SD) ratio of ascr#2 to ascr#3, for lines JU1409, JU1410, MY1, N2 and PX174.

We measured the concentration of ascr#2 and ascr#3 in these natural pheromone mixtures. This showed that the concentration of ascr#3 produced *per* worm in these cultures was significantly different among the lines (F_4,10_ = 4.5, p = 0.025) and that line MY1 produced a significantly higher concentration than that of N2 (t = 3.1, p = 0.01) (Figure 4). The concentration of ascr#2 produced *per* worm did not differ among the lines (F_4,10_ = 0.38, p = 0.81). Because ascr#2 and ascr#3 have combinatorial effects we were also interested in the ratio of these ascarosides that the lines produced. Overall this ratio did not differ significantly among the lines (F_4,10_ = 2.0, p = 0.17), though that of N2 was significantly greater than that of MY1 (t = -2.6, p = 0.02) (Figure 4). Recently, *C. elegans* strains have been found to differ in the ratio of different ascaroside molecules that they produce, for example ascr#1 (which has a very low dauer-inducing phenotype) was found to differ most among four *C. elegans* strains [20].

Together, these results mean that among these *C. elegans* lines there is (i) genetic diversity for the sensation and/or transduction of environmental ascaroside and food signals in inducing dauer larva formation and (ii) variation in the dauer-inducing signals that are produced. The dauer larva formation phenotype of the wild-type N2 is, then, just one example of many possible responses among *C. elegans* lines. This diversity of responses among these lines recently derived from the wild falsifies the idea that ascarosides are general, honest signals of conspecific population density that can be used by all lines. Rather, different *C. elegans* lines appear to have unique responses to their environmental ascaroside and food conditions.

### (c) Roles of ascarosides in *C. elegans* ecology

The combinatorial effect of ascarosides in dauer larvae formation, as well in other life history components, including dauer larva dispersal (by a four molecule mix, including ascr#2 and ascr#3) and male specific attraction, is well known [8-11,13,21-23]. Together this suggests the idea that ascarosides may be signals that are broadcast into the environment, but which can only be correctly interpreted by relatives or by ecological co-inhabitants in a specific scenario or environmental context [17].

We tested whether the genetic relatedness among the lines, measured as the average genetic distance across 50 randomly selected genes, was related to the lines’ dauer formation phenotypes when exposed to the synthetic ascarosides and food conditions, but found no significant relationships (ascr#2 2 and 5% food ρ = -0.03, p = 0.6, ρ = -0.01, p = 0.9; ascr#3 2 and 5% food ρ = 0.01, p = 0.8, ρ = -0.05, p = 0.5; ascr#2 + ascr#3 2 and 5% food ρ = -0.08, p = 0.2, ρ = 0.06, p = 0.3). While there is no such relationship with genome-wide measures of relatedness, lines’ relationships at specific loci, for example within regions previously identified as controlling dauer larvae formation sensitivity [24] may play an important key role.

In *C. elegans* a strategy of private signalling would allow a genotype to better exploit a patch of food resource, than if it had to share it with other genotypes. The natural history of *C. elegans* may promote this because as ephemeral food sources are exploited by a few dauer larvae, this may result in small, local clonal (or semi-clonal) populations [6]. There is support for this idea from other systems. For example, in the beetle *Phaedon cochleariae* components of its pheromone that is used for mate - mate contact depends on individual beetle’s food source, thus promoting mating within ecological co-inhabitants [25].

## Conclusions

For *C. elegans* the ascarosides and food in its environment modulates dauer larva formation, as well as dauer larval dispersal and male specific attraction. We have found a very substantial diversity of ascaroside signalling, with different lines producing different signals, and lines differing in how they respond to the same signal. We also find that ascr#3 promotes dauer larvae formation in some lines and represses it in others, thus showing that it is not a universal promoter of dauer larva formation. Together these findings are consistent with the idea that while ascaroside signalling is publically broadcast, correct interpretation of that signal may be semi-private among ecological co-inhabitants or relatives, or may in some other way depend on some other aspect of the worms’ context. Ascaroside signals may also be dishonest signals that worms produce because we find situations where some lines induces others, but not themselves, to form dauer larvae. Ascaroside signalling, both broadcast and receipt, using a combinatorial system of different ascaroside molecules, is therefore a dynamic and rich source of information that affects many aspects of *C. elegans* ecology.

## Methods

### (a) Worms

We used 20 *C. elegans* isolates recently isolated from the wild: JU1400, JU1401, JU1409, JU1410, JU1411, JU1416, JU1442, JU1494, obtained from Marie-Anne Felix (Institute of Biology of the Ecole Normale Supérieure, Paris); CB4853, MY1, MY2, MY16, JU262, JU319, JU345, JU362, JU393, JU400, PX174, PX179, as well as the Bristol N2 strain, obtained from the *Caenorhabditis* Genetics Centre (CGC). Worms were fed on an *Escherichia coli* OP50 food source also obtained from CGC. For each isolate, one isogenic line (referred to here as a line) was made by single-worm inbreeding for at least 10 generations (inbreeding coefficient > 0.9) and cryopreserved. Each experiment used a new cryopreserved stock of each line. The *E. coli* OP50 was cultured as previously described [26].

### (b) Dauer larva formation assay

Dauer larva formation assays were carried out as previously described [16] with all assays using 30 mm diameter plates containing with 2 mL of dauer agar [16,20]. Each dauer agar plate was inoculated with 20 μL of 2 or 5% food (w/v) of *E. coli* OP50 [24], onto which five hermaphrodites of similar age were introduced and allowed to lay eggs for a period of 3-4 hours or until approximately 50 eggs were present on each plate, after which the hermaphrodites were removed. These plates were incubated at 25°C for 48 hours, after which the dauer/non-dauer larva formation phenotype was determined. We exposed young larvae to three synthetic pheromone treatments: (i) ascr#2, (ii) ascr#3 and (iii) an equal mixture of ascr#2 and ascr#3. In these assays the final concentration of ascr#2 was 20 × 10^3^ nM and of ascr#3 66 × 10^3^ nM in both the single and mixture treatments. For the natural pheromone mixtures (below) these were used at a final concentration of 1% v/v. Each experiment (*i.e.* combination of synthetic ascarosides or natural pheromone mixture, and food treatments) was replicated three times, *i.e.* there were three assay plates. In each ascaroside or pheromone treatment and food combination we tested a different group of individual worms for each line. In order to assay all 21 lines in each experiment, we used a block design, in which we randomly allocated the 21 lines among three blocks (see (f) Data analysis, below).

### (c) Synthesis of ascr#2 and ascr#3

The overall synthetic strategy was: dibenzoate **1** was synthesised from a commercial sample of L-rhamnose, via the lactone **S** (this also being employed as an HPLC-MS standard **S**) through modifications to the procedure of [27] as follows: in the first step DMAP and Et_3_N in THF were used instead of neat pyridine, and in the fourth step DBU/DCM at -78°C was used instead of Et_3_N/chloroform at ambient temperature. ascr#2 was synthesized from **1**, following the procedure of [9]. Alkenyl ether **2** was then prepared as described in [27] from dibenzoate **1**. Ruthenium-catalyzed cross-metathesis with acrylic acid, followed by standard benzoate deprotection of the resulting dibenzoate protected enoic acid gave pheromone ascr#3. This synthetic scheme is shown in Additional file 1, together with further details of the synthesis.

### (d) Natural pheromone mixtures for use in dauer assays

We collected supernatants of liquid cultures of PX174, JU1409, JU1410, MY1 and N2 and tested their dauer larva formation phenotypes. To produce these supernatants, we grew each of these lines in 12 L of S media [26] which were fed once a week with 50 mL of 20% w/v *E. coli* OP50 in S medium *per* litre of culture, shaken at 25°C for two weeks or until the cultures had reached a density of *c.* 2,000 worms/L, which were principally L4 stages and adult hermaphrodites. The cultures were initiated with a synchronous population of *c.*1,000 starved L1s. The pheromone purification protocol was derived from [28]; specifically, at time of harvest, we filtered the supernatant through muslin to remove large debris, and then centrifuged this at 10,000 *g*, the supernatant was dried by rotary evaporation at 50°C, the dried residue extracted at least five times with 95% v/v ethanol, and this ethanol extract dried at 50°C under vacuum, and the final dried extract re-suspended in 5 mL of distilled water *per* litre of starting culture, and sterilised by filtration (0.2 μm pore size) and stored at 4°C.

### (e) Natural pheromone mixtures for quantification of ascr#2 and ascr#3

We collected supernatants of liquid cultures of PX174, JU1409, JU1410, MY1 and N2, as above, except that each culture was initiated with a synchronous population of *c*.10,000 starved L1s and the culture was grown until it had reached a density of *c*.20 million worms/L, when it was harvested, at which time we also recorded the number of L4 and adult hermaphrodite stages present. There were three 1 L replicates *per* line. The pheromone mixture was evaporated to dryness using a rotary evaporator; trituration in methanol followed by re-evaporation yielded free-flowing powders of known mass. Weighed samples of the powder were suspended in methanol to extract the pheromones, the mixture passed through micron-filtration discs to remove insoluble buffer salts *etc*. The resulting solutions, following sequential dilution, were analysed by HPLC-MS. Further details of the HPLC-MS analysis, sample dilution and the construction of calibration curves are described in Additional file 1. For each line we obtained three LC-MS readings for each replicate.

### (f) Data analysis

We used Generalised Linear Mixed-Effects Models (GLMM) to investigate the variation in dauer larvae formation between Lines, Synthetic ascarosides or Natural pheromone mixtures and Food concentration treatment. We used the logit function with a binomial distribution [29] to describe the proportion of dauer larvae (*p*) and arrested L4 non-dauer larvae (*q)* among lines, ascarosides/pheromone and food treatments, and sample size (*n*) per plate. The analysis was individually conducted for each pheromone type (*i.e.*Synthetic and Natural). For the synthetic ascarosides, the models were constructed to contrast the response of the mixture (ascr#2 + ascr#3) to the response of each ascaroside individually, and for the natural pheromone mixtures, the models compared the response of each natural pheromone mixture to that of N2. For model construction, we started with the simplest null model that included only the overall mean, and then we added explanatory variables and their interactions sequentially. We tested the goodness of fit using log-likelihood ratio tests (LRT) between nested models. Model comparison included degrees of freedom (d.f.), χ^2^ value, and p value [29]. Model comparison and results are presented in Additional file 1. To account for the experimental design, a block effect was included in each model as a random effect.

Analyses were performed using R software (version 2.13.1, R-project). Unless otherwise stated data are shown as the mean ± standard error of the mean. The data presented for each worm line are the mean of the three replicates.

For the heat map analysis, for each data set (*i.e.* the mean dauer formation phenotype (i) across 6 environments for each line, (ii) across all lines for each of the 6 environments) the Euclidean distance was calculated and then these were clustered hierarchically using complete linkage in R.

For the data on the concentration of ascr#2 and ascr#3 produced by lines PX174, JU1409, JU1410, MY1 and N2, for each line we calculated the mean value across the three LC-MS readings of each replicate. We then calculated (i) the mean concentration of each ascaroside *per* worm and from this (ii) the ascr#2/ascr#3 ratio for each replicate separately. We then used GLMs to compare these concentrations and ratios among the five lines.

### (g) Genomic DNA sequence and bioinformatic analysis

Whole genome sequence for all the lines, except CB4853, was determined by the NERC Bimolecular Analysis Facility, Edinburgh. TruSeq (Illumina) paired-end libraries were constructed following the manufacturer’s instructions, sequenced on HiSeq2000and processed using the Illumina pipeline 1.6. Sequence reads from each line were aligned to the *C. elegans* genome WS220 with alignment errors corrected using GATK; SNPs were called and their effect predicted using SamTools 0.1.18 and SnpEff 2.0.4, respectively, and from which a consensus genome for each line was constructed. We wished to estimate the genetic distance among the lines, which we did by randomly selecting 50 genes whose coding sequence was extracted from the consensus genome sequence of each line with WormMart (0.7) and BEDTools (2.17.0) as further described in Additional file 1. For each line these sequences were concatenated and then all 21 such concatenated sequences were aligned and the genetic distance calculated using MEGA5 [30] as further described in Additional file 1. We sought correlations, using Spearman’s rank correlation (ρ), between the genetic distances among each of the lines and the dauer larva formation phenotypic distance among each of the lines, considering each of the six environmental conditions separately.

## Availability of supporting data

The sequence data arising from the Methods section (g) and forming Additional file 1 is available as nebc.nerc.ac.uk:nebcfs:Viney/Additional_Files_section_4.txt at the NERC Environmental Bioinformatics Centre (NEBC) at http://nebc.nerc.ac.uk/nebcfs/public/Viney/Additional_Files_section_4.txt.

## Abbreviations

CGC: *Caenorhabditis* genetics center; GLMM: Generalised linear mixed-effects models; LRT: Likelihood ratio tests; SD: Standard deviation; HPLC-MS: High performance liquid chromatography-mass spectrometry; SNPs: Single nucleotide polymorphisms; DMAP: 4-N,N-dimethylaminopyridine; THF: Tetrahydrofuran; DBU: 1,8-diazabicycloundec-7-ene; DCM: Dichloromethane.

## Competing interests

The authors declare that they have no competing interests.

## Authors’ contributions

AD designed and executed the worm experiments, analysed and interpreted the data, and contributed to the manuscript; VB undertook the ascaroside synthesis and ascaroside quantification, GL-J determined the ascaroside synthesis strategy, data interpretation, and contributed to the manuscript; WS and BW contributed to the bioinformatic analyses; MEV contributed to the experimental design, contributed to the analysis and interpretation of the data, and led the writing of the manuscript. All authors read and approved the final manuscript.

## Supplementary Material

Additional file 1 GLMM results, dauer larva formation data, genetic analyses (section 1), ascaroside synthesis (section 2), ascaroside quantification (section 3) and sequence information (section 4). Click here for file
