# Supplementary material for: Diverse and potentially manipulative signalling with ascarosides in the model nematode C. elegans
Source: BMC Evol Biol. 2014 Mar 11;14:46. doi: 10.1186/1471-2148-14-46 (PMC4007702; doi:10.1186/1471-2148-14-46)
Supplement: Additional file 1 — GLMM results, dauer larva formation data, genetic analyses (section 1), ascaroside synthesis (section 2), ascaroside quantification (section 3) and sequence information (section 4). [file 1471-2148-14-46-S1.docx]

**Additional Files. GLMM results, dauer larva formation data, genetic analyses (section 1), ascaroside synthesis (section 2), ascaroside quantification (section 3) and sequence information (section 4)**

**Additional Files section 1. Tables 1-5 GLMM results; Table 6 dauer larva formation data; Table 7-8 genetic analyses**

**Additional File Table 1.** Generalised Linear Mixed-Effect Model (GLMM) selection describing the dauer larvae response in relation to Line, Synthetic and Food variables, showing the explanatory variables in the model, the number of parameters (K), the Akaike Informaiton Criterion (AIC) value, χ^2^ value of the LRT, and the associated p value. The best model is shown in bold. Note, the K number includes the random term of the block effect.

| **Models** | **Explanatory variables** | **K** | **AIC** | **χ^2^** | **p value** |
| --- | --- | --- | --- | --- | --- |
| M1 | Null | 2 | 9754.3 |  |  |
| M2 | Line | 22 | 8164.1 | 1629.70 | <0.001 |
| M3 | Line + Synthetic | 24 | 8152.7 | 15.88 | <0.001 |
| M4 | Line * Synthetic | 64 | 5125.4 | 3107.3 | <0.001 |
| M5 | Line * Synthetic + Food | 65 | 5083.2 | 44.20 | <0.001 |
| M6 | Line * Synthetic + Line* Food | 85 | 4510.9 | 612.34 | <0.001 |
| M7 | Line * Synthetic + Line* Food + Synthetic * Food | 87 | 3830.4 | 684.47 | <0.001 |
| **M8** | **Line * Synthetic * Food** | **127** | **3032.4** | **877.98** | **<0.001** |

**Additional File Table 2.** Results of the GLMM describing the parameters of Model 8 from Table 1. We used a binomial error distribution with logit function to describe the proportion of dauer larvae across groups. The table describes the fixed parameters, the estimates, standard error (SE), z value, corresponding p value and, at the end of the table, the random effects. There were 378 observations grouped into 9 blocks.

| **Fixed effects** | **Estimate** | **SE** | **z value** | **p value** |
| --- | --- | --- | --- | --- |
| (Intercept) | 0.51 | 0.18 | 2.94 | <0.01 |
| CB4853 | -4.69 | 1.02 | -4.59 | <0.001 |
| JU1400 | -2.00 | 0.32 | -6.23 | <0.001 |
| JU1401 | -2.05 | 0.28 | -7.28 | <0.001 |
| JU1409 | -1.05 | 0.30 | -3.50 | <0.001 |
| JU1410 | -0.47 | 0.27 | -1.79 | 0.07 |
| JU1411 | -1.68 | 0.30 | -5.68 | <0.001 |
| JU1416 | 0.69 | 0.27 | 2.55 | 0.01 |
| JU1442 | -1.84 | 0.41 | -4.46 | <0.001 |
| JU1494 | -2.13 | 0.30 | -7.20 | <0.001 |
| JU262 | -2.65 | 0.33 | -7.98 | <0.001 |
| JU319 | -1.90 | 0.33 | -5.76 | <0.001 |
| JU345 | 0.73 | 0.26 | 2.79 | <0.01 |
| JU362 | -0.51 | 0.26 | -1.96 | 0.05 |
| JU393 | 0.56 | 0.24 | 2.31 | <0.05 |
| JU400 | -1.72 | 0.25 | -6.79 | <0.001 |
| MY1 | -1.13 | 0.25 | -4.52 | <0.001 |
| MY16 | -3.81 | 0.49 | -7.81 | <0.001 |
| MY2 | 0.43 | 0.25 | 1.70 | 0.09 |
| PX174 | -4.04 | 0.54 | -7.53 | <0.001 |
| PX179 | -5.07 | 0.73 | -6.93 | <0.001 |
| ascr#2 | -0.66 | 0.23 | -2.91 | <0.01 |
| ascr#3 | -2.41 | 0.24 | -10.21 | <0.001 |
| Food5% | -1.36 | 0.26 | -5.16 | <0.001 |
| CB4853:ascr#2 | 1.49 | 1.15 | 1.29 | 0.20 |
| JU1400:ascr#2 | 1.30 | 0.39 | 3.36 | <0.001 |
| JU1401:ascr#2 | -0.50 | 0.45 | -1.09 | 0.27 |
| JU1409:ascr#2 | 0.02 | 0.39 | 0.04 | 0.97 |
| JU1410:ascr#2 | -0.28 | 0.33 | -0.86 | 0.39 |
| JU1411:ascr#2 | 1.08 | 0.36 | 3.01 | <0.01 |
| JU1416:ascr#2 | 0.07 | 0.34 | 0.21 | 0.83 |
| JU1442:ascr#2 | 1.37 | 0.51 | 2.66 | <0.01 |
| JU1494:ascr#2 | 1.18 | 0.37 | 3.23 | <0.01 |
| JU262:ascr#2 | 2.76 | 0.41 | 6.67 | <0.001 |
| JU319:ascr#2 | 1.83 | 0.39 | 4.65 | <0.001 |
| JU345:ascr#2 | 0.66 | 0.36 | 1.85 | 0.06 |
| JU362:ascr#2 | 1.86 | 0.34 | 5.46 | <0.001 |
| JU393:ascr#2 | 1.11 | 0.32 | 3.47 | <0.001 |
| JU400:ascr#2 | 1.74 | 0.33 | 5.20 | <0.001 |
| MY1:ascr#2 | 1.13 | 0.33 | 3.44 | <0.001 |
| MY16:ascr#2 | 0.40 | 0.78 | 0.51 | 0.61 |
| MY2:ascr#2 | -0.36 | 0.31 | -1.15 | 0.25 |
| PX174:ascr#2 | 0.15 | 0.75 | 0.20 | 0.84 |
| PX179:ascr#2 | 2.19 | 0.77 | 2.85 | <0.01 |
| CB4853:ascr#3 | 4.75 | 1.08 | 4.41 | <0.001 |
| JU1400:ascr#3 | 0.55 | 0.49 | 1.12 | 0.26 |
| JU1401:ascr#3 | 4.83 | 0.36 | 13.24 | <0.001 |
| JU1409:ascr#3 | -1.12 | 0.53 | -2.09 | <0.05 |
| JU1410:ascr#3 | 0.49 | 0.42 | 1.19 | 0.24 |
| JU1411:ascr#3 | 4.18 | 0.39 | 10.61 | <0.001 |
| JU1416:ascr#3 | -0.14 | 0.37 | -0.38 | 0.71 |
| JU1442:ascr#3 | 1.69 | 0.50 | 3.38 | <0.001 |
| JU1494:ascr#3 | 0.87 | 0.57 | 1.53 | 0.13 |
| JU262:ascr#3 | 5.63 | 0.40 | 13.99 | <0.001 |
| JU319:ascr#3 | 3.25 | 0.43 | 7.49 | <0.001 |
| JU345:ascr#3 | -1.05 | 0.39 | -2.72 | <0.01 |
| JU362:ascr#3 | 1.62 | 0.33 | 4.89 | <0.001 |
| JU393:ascr#3 | -0.61 | 0.37 | -1.65 | 0.10 |
| JU400:ascr#3 | -0.44 | 0.48 | -0.91 | 0.36 |
| MY1:ascr#3 | 2.45 | 0.37 | 6.66 | <0.001 |
| MY16:ascr#3 | 0.92 | 0.77 | 1.19 | 0.23 |
| MY2:ascr#3 | -0.75 | 0.35 | -2.12 | <0.05 |
| PX174:ascr#3 | 4.06 | 0.94 | 4.31 | <0.001 |
| PX179:ascr#3 | 7.37 | 0.76 | 9.67 | <0.001 |
| CB4853:Food5% | 3.73 | 1.07 | 3.50 | <0.001 |
| JU1400:Food5% | 1.02 | 0.45 | 2.28 | <0.05 |
| JU1401:Food5% | 3.65 | 0.38 | 9.59 | <0.001 |
| JU1409:Food5% | 2.75 | 0.39 | 7.07 | <0.001 |
| JU1410:Food5% | 1.71 | 0.36 | 4.73 | <0.001 |
| JU1411:Food5% | 2.00 | 0.39 | 5.13 | <0.001 |
| JU1416:Food5% | -0.72 | 0.37 | -1.92 | 0.05 |
| JU1442:Food5% | 1.65 | 0.49 | 3.39 | <0.001 |
| JU1494:Food5% | 2.93 | 0.38 | 7.67 | <0.001 |
| JU262:Food5% | 1.69 | 0.46 | 3.68 | <0.001 |
| JU319:Food5% | 1.13 | 0.45 | 2.52 | <0.05 |
| JU345:Food5% | 0.63 | 0.36 | 1.77 | 0.08 |
| JU362:Food5% | 2.03 | 0.36 | 5.62 | <0.001 |
| JU393:Food5% | -0.13 | 0.35 | -0.37 | 0.71 |
| JU400:Food5% | 1.16 | 0.38 | 3.08 | <0.01 |
| MY1:Food5% | 1.15 | 0.37 | 3.14 | <0.01 |
| MY16:Food5% | 4.63 | 0.55 | 8.46 | <0.001 |
| MY2:Food5% | 0.66 | 0.35 | 1.90 | 0.06 |
| PX174:Food5% | 4.02 | 0.61 | 6.62 | <0.001 |
| PX179:Food5% | 3.04 | 0.87 | 3.50 | <0.001 |
| ascr#2:Food5% | -3.04 | 0.77 | -3.94 | <0.001 |
| ascr#3:Food5% | 3.64 | 0.34 | 10.84 | <0.001 |
| CB4853:ascr#2:Food5% | 0.32 | 1.50 | 0.22 | 0.83 |
| JU1400:ascr#2:Food5% | -0.75 | 1.33 | -0.56 | 0.57 |
| JU1401:ascr#2:Food5% | 0.71 | 0.91 | 0.78 | 0.44 |
| JU1409:ascr#2:Food5% | 0.00 | 0.90 | 0.00 | 1.00 |
| JU1410:ascr#2:Food5% | 1.58 | 0.84 | 1.89 | 0.06 |
| JU1411:ascr#2:Food5% | 1.79 | 0.85 | 2.12 | <0.05 |
| JU1416:ascr#2:Food5% | 0.97 | 1.01 | 0.96 | 0.34 |
| JU1442:ascr#2:Food5% | 1.40 | 0.99 | 1.42 | 0.16 |
| JU1494:ascr#2:Food5% | 1.25 | 0.86 | 1.46 | 0.14 |
| JU262:ascr#2:Food5% | 1.10 | 0.90 | 1.21 | 0.22 |
| JU319:ascr#2:Food5% | 1.56 | 0.90 | 1.74 | 0.08 |
| JU345:ascr#2:Food5% | -0.10 | 0.86 | -0.11 | 0.91 |
| JU362:ascr#2:Food5% | 2.62 | 0.87 | 3.03 | <0.01 |
| JU393:ascr#2:Food5% | 0.84 | 0.84 | 1.00 | 0.32 |
| JU400:ascr#2:Food5% | 2.14 | 0.85 | 2.52 | <0.05 |
| MY1:ascr#2:Food5% | 1.92 | 0.85 | 2.26 | <0.05 |
| MY16:ascr#2:Food5% | 2.02 | 1.11 | 1.82 | 0.07 |
| MY2:ascr#2:Food5% | 2.32 | 0.83 | 2.79 | <0.01 |
| PX174:ascr#2:Food5% | -0.19 | 1.18 | -0.16 | 0.88 |
| PX179:ascr#2:Food5% | 1.85 | 1.16 | 1.60 | 0.11 |
| CB4853:ascr#3:Food5% | -4.62 | 1.13 | -4.08 | <0.001 |
| JU1400:ascr#3:Food5% | -3.99 | 0.93 | -4.28 | <0.001 |
| JU1401:ascr#3:Food5% | -5.57 | 0.51 | -11.01 | <0.001 |
| JU1409:ascr#3:Food5% | -6.18 | 0.93 | -6.64 | <0.001 |
| JU1410:ascr#3:Food5% | -2.14 | 0.53 | -4.07 | <0.001 |
| JU1411:ascr#3:Food5% | -4.62 | 0.52 | -8.83 | <0.001 |
| JU1416:ascr#3:Food5% | 0.50 | 0.51 | 0.99 | 0.32 |
| JU1442:ascr#3:Food5% | -2.94 | 0.60 | -4.87 | <0.001 |
| JU1494:ascr#3:Food5% | -2.55 | 0.64 | -3.95 | <0.001 |
| JU262:ascr#3:Food5% | -4.01 | 0.55 | -7.28 | <0.001 |
| JU319:ascr#3:Food5% | -3.95 | 0.57 | -6.91 | <0.001 |
| JU345:ascr#3:Food5% | -1.71 | 0.54 | -3.18 | <0.05 |
| JU362:ascr#3:Food5% | -7.13 | 0.60 | -11.87 | <0.001 |
| JU393:ascr#3:Food5% | -0.10 | 0.49 | -0.20 | 0.84 |
| JU400:ascr#3:Food5% | -1.95 | 0.64 | -3.03 | <0.05 |
| MY1:ascr#3:Food5% | -3.18 | 0.50 | -6.31 | <0.001 |
| MY16:ascr#3:Food5% | -5.38 | 0.88 | -6.12 | <0.001 |
| MY2:ascr#3:Food5% | -1.78 | 0.46 | -3.83 | <0.001 |
| PX174:ascr#3:Food5% | -5.20 | 1.01 | -5.15 | <0.001 |
| PX179:ascr#3:Food5% | -5.80 | 0.91 | -6.38 | <0.001 |
|  |  |  |  |  |
| **Random effects** |  | **Group name** | **Variance** | **Standard**  **Deviation** |
| Block |  | Intercept | 1.73e-13 | 4.16e-7 |

**Additional File Table 3.** The mean and SE of dauer formation of the 21 *C. elegans* lines in synthetic ascaroside environments with 2% and 5% food conditions.

|  | **2% food** | | | | | | **5% food** | | | | | |
| --- | --- | --- | --- | --- | --- | --- | --- | --- | --- | --- | --- | --- |
|  | **ascr#2** | | **ascr#3** | | **ascr#2 + #3** | | **ascr#2** | | **ascr#3** | | **ascr#2 + #3** | |
|  | Mean | SE | Mean | SE | Mean | SE | Mean | SE | Mean | SE | Mean | SE |
| N2 | 0.47 | 0.03 | 0.16 | 0.08 | 0.61 | 0.02 | 0.01 | 0.01 | 0.55 | 0.17 | 0.31 | 0.05 |
| CB4853 | 0.04 | 0.02 | 0.13 | 0.05 | 0.02 | 0.02 | 0.02 | 0.02 | 0.38 | 0.04 | 0.14 | 0.07 |
| JU1400 | 0.31 | 0.06 | 0.05 | 0.02 | 0.19 | 0.02 | 0.01 | 0.01 | 0.02 | 0.01 | 0.16 | 0.06 |
| JU1401 | 0.05 | 0.03 | 0.73 | 0.12 | 0.16 | 0.05 | 0.06 | 0.01 | 0.78 | 0.02 | 0.66 | 0.13 |
| JU1409 | 0.21 | 0.11 | 0.02 | 0.00 | 0.42 | 0.20 | 0.10 | 0.10 | 0.00 | 0.00 | 0.72 | 0.08 |
| JU1410 | 0.31 | 0.09 | 0.11 | 0.06 | 0.49 | 0.07 | 0.11 | 0.06 | 0.48 | 0.07 | 0.60 | 0.03 |
| JU1411 | 0.33 | 0.05 | 0.66 | 0.10 | 0.21 | 0.08 | 0.20 | 0.07 | 0.57 | 0.05 | 0.38 | 0.02 |
| JU1416 | 0.64 | 0.05 | 0.21 | 0.02 | 0.78 | 0.06 | 0.02 | 0.02 | 0.66 | 0.07 | 0.32 | 0.10 |
| JU1442 | 0.39 | 0.27 | 0.11 | 0.06 | 0.20 | 0.09 | 0.19 | 0.12 | 0.22 | 0.10 | 0.22 | 0.12 |
| JU1494 | 0.24 | 0.08 | 0.05 | 0.04 | 0.17 | 0.03 | 0.21 | 0.15 | 0.41 | 0.09 | 0.48 | 0.07 |
| JU262 | 0.41 | 0.14 | 0.74 | 0.08 | 0.15 | 0.10 | 0.16 | 0.04 | 0.71 | 0.17 | 0.13 | 0.11 |
| JU319 | 0.44 | 0.17 | 0.40 | 0.09 | 0.22 | 0.06 | 0.13 | 0.07 | 0.27 | 0.04 | 0.16 | 0.04 |
| JU345 | 0.78 | 0.04 | 0.10 | 0.06 | 0.78 | 0.03 | 0.06 | 0.02 | 0.34 | 0.12 | 0.65 | 0.10 |
| JU362 | 0.73 | 0.06 | 0.31 | 0.03 | 0.52 | 0.15 | 0.78 | 0.08 | 0.04 | 0.03 | 0.71 | 0.09 |
| JU393 | 0.82 | 0.03 | 0.13 | 0.03 | 0.78 | 0.09 | 0.10 | 0.03 | 0.53 | 0.03 | 0.40 | 0.02 |
| JU400 | 0.47 | 0.05 | 0.01 | 0.01 | 0.24 | 0.08 | 0.23 | 0.04 | 0.05 | 0.04 | 0.19 | 0.05 |
| MY1 | 0.46 | 0.02 | 0.39 | 0.19 | 0.38 | 0.17 | 0.19 | 0.03 | 0.42 | 0.09 | 0.28 | 0.09 |
| MY16 | 0.02 | 0.02 | 0.01 | 0.00 | 0.03 | 0.02 | 0.13 | 0.08 | 0.04 | 0.01 | 0.38 | 0.21 |
| MY2 | 0.49 | 0.04 | 0.15 | 0.10 | 0.73 | 0.11 | 0.20 | 0.04 | 0.29 | 0.06 | 0.59 | 0.08 |
| PX179 | 0.05 | 0.01 | 0.61 | 0.07 | 0.01 | 0.01 | 0.07 | 0.01 | 0.52 | 0.14 | 0.07 | 0.07 |
| PX174 | 0.02 | 0.01 | 0.20 | 0.15 | 0.03 | 0.01 | 0.01 | 0.01 | 0.32 | 0.12 | 0.27 | 0.13 |

**Additional File Table 4.** Generalised Linear Mixed-Effect Model (GLMM) selection describing the dauer larvae response in relation to Line, Natural and Food variables, showing the explanatory variables in the model, the number of parameters (K), the AIC value, χ^2^ value of the LRT, and the associated p value. The best model is shown in bold. Note, the K number includes the random term of the block effect.

| **Models** | **Explanatory variables** | **K** | **AIC** | **χ^2^** | **p value** |
| --- | --- | --- | --- | --- | --- |
| M1 | Null | 2 | 15430 |  |  |
| M2 | Line | 22 | 12883 | 2586.7 | <0.001 |
| M3 | Line + Natural | 26 | 12876 | 15.5 | <0.01 |
| M4 | Line * Natural | 106 | 7628 | 5407.4 | <0.001 |
| M5 | Line * Natural + Food | 107 | 7556 | 74.2 | <0.001 |
| M6 | Line * Natural + Line* Food | 127 | 6644 | 951.7 | <0.001 |
| M7 | Line * Natural + Line* Food + Natural * Food | 131 | 6555 | 96.4 | <0.001 |
| **M8** | **Line * Natural * Food** | **211** | **5422** | **1293.8** | **<0.001** |

**Additional File Table 5.** Results of the GLMM describing the parameters of Model 8 from Table 4. We used a binomial error distribution with logit function to describe the proportion of dauer larvae across groups. The table describes the fixed parameters, the estimates, standard error (SE), z value, corresponding p value and, at the end of the table, the random effects. There were 630 observations grouped into 15 blocks.

| **Fixed effects** | **Estimate** | **SE** | **z value** | **p value** |
| --- | --- | --- | --- | --- |
| (Intercept) | 1.15 | 0.16 | 7.28 | <0.001 |
| CB4853 | -4.25 | 0.53 | -7.95 | <0.001 |
| JU1400 | -1.03 | 0.19 | -5.39 | <0.001 |
| JU1401 | -0.63 | 0.20 | -3.23 | <0.01 |
| JU1409 | -3.42 | 0.21 | -16.56 | <0.001 |
| JU1410 | -0.89 | 0.22 | -4.05 | <0.001 |
| JU1411 | -1.12 | 0.22 | -5.11 | <0.001 |
| JU1416 | -0.99 | 0.22 | -4.57 | <0.001 |
| JU1442 | 1.60 | 0.45 | 3.57 | <0.001 |
| JU1494 | -2.96 | 0.28 | -10.43 | <0.001 |
| JU262 | -1.86 | 0.21 | -8.67 | <0.001 |
| JU319 | -1.09 | 0.19 | -5.80 | <0.001 |
| JU345 | -2.87 | 0.44 | -6.52 | <0.001 |
| JU362 | 0.13 | 0.24 | 0.56 | 0.58 |
| JU393 | 0.98 | 0.41 | 2.41 | <0.05 |
| JU400 | -0.85 | 0.20 | -4.22 | <0.001 |
| MY1 | 0.98 | 0.28 | 3.57 | <0.001 |
| MY16 | -3.20 | 0.23 | -13.96 | <0.001 |
| MY2 | -3.85 | 0.33 | -11.78 | <0.001 |
| PX174 | -0.55 | 0.19 | -2.93 | <0.01 |
| PX179 | -1.23 | 0.18 | -6.71 | <0.001 |
| Pher 5 | -1.26 | 0.29 | -4.40 | <0.001 |
| Pher 6 | -1.18 | 0.24 | -4.96 | <0.001 |
| pher17 | -2.72 | 0.27 | -10.18 | <0.001 |
| pher20 | -3.49 | 0.43 | -8.20 | <0.001 |
| Food5% | -1.97 | 0.20 | -9.61 | <0.001 |
| CB4853:PherJU1409 | 2.77 | 0.65 | 4.25 | <0.001 |
| JU1400:PherJU1409 | -0.61 | 0.42 | -1.45 | 0.15 |
| JU1401:PherJU1409 | 1.33 | 0.40 | 3.32 | <0.001 |
| JU1409:PherJU1409 | 1.86 | 0.34 | 5.43 | <0.001 |
| JU1410:PherJU1409 | 0.75 | 0.35 | 2.15 | <0.05 |
| JU1411:PherJU1409 | 0.69 | 0.37 | 1.88 | 0.06 |
| JU1416:PherJU1409 | 1.30 | 0.35 | 3.73 | <0.001 |
| JU1442:PherJU1409 | -3.79 | 0.57 | -6.65 | <0.001 |
| JU1494:PherJU1409 | 2.24 | 0.41 | 5.50 | <0.001 |
| JU262:PherJU1409 | 1.56 | 0.34 | 4.52 | <0.001 |
| JU319:PherJU1409 | -2.09 | 1.06 | -1.97 | <0.05 |
| JU345:PherJU1409 | -17.86 | 1472.00 | -0.01 | 0.99 |
| JU362:PherJU1409 | 0.59 | 0.38 | 1.55 | 0.12 |
| JU393:PherJU1409 | -2.05 | 0.56 | -3.68 | <0.001 |
| JU400:PherJU1409 | 0.00 | 0.36 | 0.01 | 0.99 |
| MY1:PherJU1409 | -1.80 | 0.40 | -4.51 | <0.001 |
| MY16:PherJU1409 | 1.07 | 0.42 | 2.52 | <0.05 |
| MY2:PherJU1409 | 3.61 | 0.50 | 7.29 | <0.001 |
| PX174:PherJU1409 | -1.66 | 0.40 | -4.14 | <0.001 |
| PX179:PherJU1409 | -0.18 | 0.41 | -0.45 | 0.65 |
| CB4853:PherJU1410 | -0.79 | 1.15 | -0.68 | 0.49 |
| JU1400:PherJU1410 | -1.09 | 0.36 | -3.07 | <0.01 |
| JU1401:PherJU1410 | -0.50 | 0.31 | -1.59 | 0.11 |
| JU1409:PherJU1410 | 0.95 | 0.48 | 1.99 | <0.05 |
| JU1410:PherJU1410 | 0.59 | 0.35 | 1.69 | 0.09 |
| JU1411:PherJU1410 | 0.63 | 0.32 | 1.98 | <0.05 |
| JU1416:PherJU1410 | 0.58 | 0.31 | 1.88 | 0.06 |
| JU1442:PherJU1410 | -3.07 | 0.54 | -5.68 | <0.001 |
| JU1494:PherJU1410 | 0.15 | 0.51 | 0.30 | 0.76 |
| JU262:PherJU1410 | 1.90 | 0.31 | 6.20 | <0.001 |
| JU319:PherJU1410 | -2.38 | 0.57 | -4.18 | <0.001 |
| JU345:PherJU1410 | -16.19 | 1507.00 | -0.01 | 0.99 |
| JU362:PherJU1410 | -19.29 | 1469.00 | -0.01 | 0.99 |
| JU393:PherJU1410 | -4.37 | 0.63 | -6.89 | <0.001 |
| JU400:PherJU1410 | -0.82 | 0.40 | -2.02 | <0.05 |
| MY1:PherJU1410 | -19.83 | 1500.00 | -0.01 | 0.99 |
| MY16:PherJU1410 | 2.31 | 0.34 | 6.87 | <0.001 |
| MY2:PherJU1410 | 0.85 | 0.70 | 1.22 | 0.22 |
| PX174:PherJU1410 | -1.98 | 0.53 | -3.73 | <0.001 |
| PX179:PherJU1410 | -0.47 | 0.42 | -1.12 | 0.26 |
| CB4853: MY1 | 4.54 | 0.60 | 7.59 | <0.001 |
| JU1400:Pher MY1 | 0.53 | 0.37 | 1.41 | 0.16 |
| JU1401:Pher MY1 | 2.18 | 0.31 | 7.02 | <0.001 |
| JU1409:Pher MY1 | 3.57 | 0.36 | 9.94 | <0.001 |
| JU1410:Pher MY1 | 1.06 | 0.36 | 2.94 | <0.01 |
| JU1411:Pher MY1 | 1.99 | 0.38 | 5.25 | <0.001 |
| JU1416:Pher MY1 | 0.77 | 0.37 | 2.06 | <0.05 |
| JU1442:Pher MY1 | -2.36 | 0.57 | -4.14 | <0.001 |
| JU1494:Pher MY1 | 2.85 | 0.42 | 6.84 | <0.001 |
| JU262:Pher MY1 | 1.46 | 0.39 | 3.73 | <0.001 |
| JU319:Pher MY1 | 0.21 | 0.42 | 0.49 | 0.63 |
| JU345:Pher MY1 | 3.57 | 0.52 | 6.93 | <0.001 |
| JU362:Pher MY1 | 1.43 | 0.34 | 4.17 | <0.001 |
| JU393:Pher MY1 | 0.19 | 0.48 | 0.40 | 0.69 |
| JU400:Pher MY1 | 1.29 | 0.35 | 3.73 | <0.001 |
| MY1:Pher MY1 | -0.86 | 0.42 | -2.05 | <0.05 |
| MY16:Pher MY1 | 3.12 | 0.38 | 8.28 | <0.001 |
| MY2:Pher MY1 | 3.14 | 0.50 | 6.23 | <0.001 |
| PX174:PherMY1 | -0.30 | 0.41 | -0.73 | 0.46 |
| PX179:PherMY1 | 0.50 | 0.47 | 1.07 | 0.28 |
| CB4853:PherPX174 | -12.14 | 1511.00 | -0.01 | 0.99 |
| JU1400:Pher PX174 | -1.27 | 0.84 | -1.52 | 0.13 |
| JU1401:Pher PX174 | 1.97 | 0.46 | 4.25 | <0.001 |
| JU1409:Pher PX174 | 3.31 | 0.49 | 6.80 | <0.001 |
| JU1410:Pher PX174 | 2.32 | 0.48 | 4.79 | <0.001 |
| JU1411:Pher PX174 | 3.22 | 0.50 | 6.47 | <0.001 |
| JU1416:Pher PX174 | 0.61 | 0.62 | 0.98 | 0.33 |
| JU1442:Pher PX174 | -1.68 | 0.76 | -2.21 | <0.05 |
| JU1494:Pher PX174 | 5.36 | 0.55 | 9.77 | <0.001 |
| JU262:Pher PX174 | 3.75 | 0.49 | 7.61 | <0.001 |
| JU319:Pher PX1749 | -15.58 | 1427.00 | -0.01 | 0.99 |
| JU345:Pher PX174 | 3.65 | 0.64 | 5.73 | <0.001 |
| JU362:Pher PX174 | 1.05 | 0.50 | 2.11 | <0.05 |
| JU393:Pher PX174 | 0.12 | 0.64 | 0.19 | 0.85 |
| JU400:Pher PX174 | 24.96 | 4318.00 | 0.01 | 1.00 |
| MY1:Pher PX174 | -1.82 | 0.87 | -2.09 | <0.05 |
| MY16:Pher PX1749 | 9.90 | 0.85 | 11.70 | <0.001 |
| MY2:Pher PX174 | 4.42 | 0.59 | 7.50 | <0.001 |
| PX174:Pher PX17409 | 1.80 | 0.53 | 3.39 | <0.001 |
| PX179:Pher PX174 | 7.53 | 1.10 | 6.85 | <0.001 |
| CB4853:Food5% | -15.32 | 1463.00 | -0.01 | 0.99 |
| JU1400:Food5% | 0.99 | 0.25 | 3.93 | <0.001 |
| JU1401:Food5% | -0.43 | 0.27 | -1.59 | 0.11 |
| JU1409:Food5% | 3.43 | 0.26 | 13.10 | <0.001 |
| JU1410:Food5% | 2.85 | 0.28 | 10.09 | <0.001 |
| JU1411:Food5% | 2.02 | 0.28 | 7.24 | <0.001 |
| JU1416:Food5% | 3.79 | 0.32 | 11.91 | <0.001 |
| JU1442:Food5% | -1.81 | 0.49 | -3.68 | <0.001 |
| JU1494:Food5% | 3.16 | 0.34 | 9.29 | <0.001 |
| JU262:Food5% | 3.21 | 0.28 | 11.41 | <0.001 |
| JU319:Food5% | 1.14 | 0.26 | 4.40 | <0.001 |
| JU345:Food5% | 3.91 | 0.57 | 6.88 | <0.001 |
| JU362:Food5% | 0.28 | 0.31 | 0.88 | 0.38 |
| JU393:Food5% | 2.71 | 0.54 | 5.05 | <0.001 |
| JU400:Food5% | 2.85 | 0.30 | 9.51 | <0.001 |
| MY1:Food5% | 0.78 | 0.35 | 2.21 | <0.05 |
| MY16:Food5% | 2.44 | 0.33 | 7.45 | <0.001 |
| MY2:Food5% | 3.41 | 0.39 | 8.73 | <0.001 |
| PX174:Food5% | 1.84 | 0.24 | 7.55 | <0.001 |
| PX179:Food5% | 1.15 | 0.27 | 4.31 | <0.001 |
| JU1409:Food5% | -2.22 | 0.78 | -2.85 | <0.01 |
| JU1410:Food5% | -0.05 | 0.36 | -0.14 | 0.89 |
| MY1:Food5% | 0.97 | 0.52 | 1.88 | 0.06 |
| PX174:Food5% | -0.64 | 1.10 | -0.59 | 0.56 |
| CB4853:PherJU1409:Food5% | 19.39 | 1463.00 | 0.01 | 0.99 |
| JU1400:PherJU1409:Food5% | 2.61 | 0.90 | 2.91 | <0.01 |
| JU1401:PherJU1409:Food5% | 2.47 | 1.00 | 2.47 | <0.05 |
| JU1409:PherJU1409:Food5% | 3.69 | 0.82 | 4.49 | <0.001 |
| JU1410:PherJU1409:Food5% | -0.47 | 0.84 | -0.57 | 0.57 |
| JU1411:PherJU1409:Food5% | 0.80 | 0.85 | 0.94 | 0.35 |
| JU1416:PherJU1409:Food5% | -1.07 | 0.84 | -1.27 | 0.21 |
| JU1442:PherJU1409:Food5% | 5.90 | 0.98 | 6.04 | <0.001 |
| JU1494:PherJU1409:Food5% | 0.87 | 0.86 | 1.01 | 0.31 |
| JU262:PherJU1409:Food5% | 2.00 | 0.82 | 2.44 | <0.05 |
| JU319:PherJU1409:Food5% | 4.35 | 1.38 | 3.16 | <0.01 |
| JU345:PherJU1409:Food5% | 17.56 | 1472.00 | 0.01 | 0.99 |
| JU362:PherJU1409:Food5% | 2.93 | 0.86 | 3.41 | <0.001 |
| JU393:PherJU1409:Food5% | 0.37 | 1.00 | 0.37 | 0.71 |
| JU400:PherJU1409:Food5% | -18.81 | 2314.00 | -0.01 | 0.99 |
| MY1:PherJU1409:Food5% | 2.56 | 0.86 | 2.96 | <0.01 |
| MY16:PherJU1409:Food5% | -17.04 | 2097.00 | -0.01 | 0.99 |
| MY2:PherJU1409:Food5% | -1.43 | 1.37 | -1.04 | 0.30 |
| PX174:PherJU1409:Food5% | 2.71 | 0.89 | 3.05 | <0.01 |
| PX179:PherJU1409:Food5% | -15.28 | 2313.00 | -0.01 | 0.99 |
| CB4853:PherJU1410:Food5% | 17.66 | 1463.00 | 0.01 | 0.99 |
| JU1400:PherJU1410:Food5% | 0.03 | 0.59 | 0.05 | 0.96 |
| JU1401:PherJU1410:Food5% | 1.87 | 0.49 | 3.83 | <0.001 |
| JU1409:PherJU1410:Food5% | -1.38 | 0.67 | -2.06 | <0.05 |
| JU1410:PherJU1410:Food5% | -0.36 | 0.49 | -0.74 | 0.46 |
| JU1411:PherJU1410:Food5% | -0.82 | 0.46 | -1.77 | 0.08 |
| JU1416:PherJU1410:Food5% | -3.76 | 0.55 | -6.88 | <0.001 |
| JU1442:PherJU1410:Food5% | 4.36 | 0.65 | 6.67 | <0.001 |
| JU1494:PherJU1410:Food5% | -1.81 | 0.73 | -2.49 | <0.05 |
| JU262:PherJU1410:Food5% | -2.89 | 0.49 | -5.89 | <0.001 |
| JU319:PherJU1410:Food5% | 0.88 | 0.82 | 1.08 | 0.28 |
| JU345:PherJU1410:Food5% | 12.97 | 1507.00 | 0.01 | 0.99 |
| JU362:PherJU1410:Food5% | 17.02 | 1469.00 | 0.01 | 0.99 |
| JU393:PherJU1410:Food5% | -1.47 | 0.96 | -1.53 | 0.13 |
| JU400:PherJU1410:Food5% | 0.27 | 0.54 | 0.51 | 0.61 |
| MY1:PherJU1410:Food5% | 16.52 | 1500.00 | 0.01 | 0.99 |
| MY16:PherJU1410:Food5% | -2.37 | 0.59 | -4.03 | <0.001 |
| MY2:PherJU1410:Food5% | -2.66 | 1.27 | -2.10 | <0.05 |
| PX174:PherJU1410:Food5% | -1.31 | 0.94 | -1.40 | 0.16 |
| PX179:PherJU1410:Food5% | -0.86 | 0.73 | -1.19 | 0.23 |
| CB4853:Pher MY1:Food5% | 12.61 | 1463.00 | 0.01 | 0.99 |
| JU1400:Pher MY1:Food5% | -0.15 | 0.67 | -0.23 | 0.82 |
| JU1401:Pher MY1:Food5% | 1.37 | 0.57 | 2.40 | <0.05 |
| JU1409:Pher MY1:Food5% | -5.43 | 1.16 | -4.68 | <0.001 |
| JU1410:Pher MY1:Food5% | -2.88 | 0.66 | -4.37 | <0.001 |
| JU1411:Pher MY1:Food5% | -3.15 | 0.75 | -4.19 | <0.001 |
| JU1416:Pher MY1:Food5% | -3.46 | 0.69 | -5.04 | <0.001 |
| JU1442:Pher MY1:Food5% | 3.05 | 0.79 | 3.85 | <0.001 |
| JU1494:Pher MY1:Food5% | -1.30 | 0.64 | -2.01 | <0.05 |
| JU262:Pher MY1:Food5% | -2.38 | 0.65 | -3.64 | <0.001 |
| JU319:Pher MY1:Food5% | 0.24 | 0.68 | 0.35 | 0.73 |
| JU345:Pher MY1:Food5% | -2.68 | 0.78 | -3.45 | <0.001 |
| JU362:Pher MY1:Food5% | 0.58 | 0.59 | 0.98 | 0.33 |
| JU393:Pher MY1:Food5% | -2.01 | 0.74 | -2.71 | <0.01 |
| JU400:Pher MY1:Food5% | -1.80 | 0.61 | -2.93 | <0.01 |
| MY1:Pher MY1:Food5% | -0.29 | 0.69 | -0.43 | 0.67 |
| MY16:Pher MY1:Food5% | -1.55 | 0.65 | -2.38 | <0.05 |
| MY2:Pher MY1:Food5% | -3.43 | 0.91 | -3.77 | <0.001 |
| PX174:PherMY1:Food5% | 0.32 | 0.65 | 0.50 | 0.62 |
| PX179:PherMY1:Food5% | -0.58 | 0.75 | -0.78 | 0.44 |
| CB4853:PherPX174:Food5% | 32.66 | 2103.00 | 0.02 | 0.99 |
| JU1400:Pher PX174:Food5% | 2.55 | 1.36 | 1.88 | 0.06 |
| JU1401:Pher PX174:Food5% | 3.69 | 1.13 | 3.27 | <0.01 |
| JU1409:Pher PX174:Food5% | 1.52 | 1.13 | 1.34 | 0.18 |
| JU1410:Pher PX174:Food5% | 0.94 | 1.13 | 0.83 | 0.41 |
| JU1411:Pher PX174:Food5% | 0.83 | 1.14 | 0.73 | 0.47 |
| JU1416:Pher PX174:Food5% | -1.18 | 1.24 | -0.95 | 0.34 |
| JU1442:Pher PX174:Food5% | 3.25 | 1.63 | 2.00 | <0.05 |
| JU1494:Pher PX174:Food5% | -5.10 | 1.54 | -3.32 | <0.001 |
| JU262:Pher PX174:Food5% | 1.91 | 1.16 | 1.64 | 0.10 |
| JU319:Pher PX1749:Food5% | 19.60 | 1427.00 | 0.01 | 0.99 |
| JU345:Pher PX174:Food5% | -1.81 | 1.28 | -1.41 | 0.16 |
| JU362:Pher PX174:Food5% | 1.45 | 1.16 | 1.25 | 0.21 |
| JU393:Pher PX174:Food5% | -0.76 | 1.27 | -0.59 | 0.55 |
| JU400:Pher PX174:Food5% | -0.05 | 6839.00 | 0.00 | 1.00 |
| MY1:Pher PX174:Food5% | 0.59 | 1.68 | 0.35 | 0.72 |
| MY16:Pher PX1749:Food5% | 17.05 | 3332.00 | 0.01 | 1.00 |
| MY2:Pher PX174:Food5% | -0.69 | 1.20 | -0.58 | 0.56 |
| PX174:Pher PX17409:Food5% | -1.76 | 1.23 | -1.42 | 0.15 |
| PX179:Pher PX174:Food5% | 18.81 | 3310.00 | 0.01 | 1.00 |
|  |  |  |  |  |
| **Random effects** |  | **Group name** | **Variance** | **Standard Deviation** |
| Block |  | Intercept | 2.33e-14 | 1.52e-7 |

**Additional File Table 6*.*** The mean and SE of dauer formation of the 21 *C. elegans* lines in natural pheromone environments (from JU1409, JU1410, MY1, N2 and PX174) with (a) 2% and (b) 5% food conditions. NA – not assayed.

**(a)**

|  | **JU1409** | | **JU1410** | | **MY1** | | **N2** | | **PX174** | |
| --- | --- | --- | --- | --- | --- | --- | --- | --- | --- | --- |
|  | Mean | SE | Mean | SE | Mean | SE | Mean | SE | Mean | SE |
| N2 | 0.40 | 0.27 | 0.49 | 0.03 | 0.18 | 0.04 | 0.80 | 0.16 | 0.10 | 0.03 |
| CB4853 | 0.13 | 0.07 | 0.01 | 0.01 | 0.21 | 0.04 | 0.06 | 0.04 | 0.00 | 0.00 |
| JU1400 | 0.15 | 0.04 | 0.11 | 0.03 | 0.11 | 0.04 | 0.58 | 0.11 | 0.01 | 0.01 |
| JU1401 | 0.44 | 0.22 | 0.24 | 0.03 | 0.47 | 0.07 | 0.76 | 0.19 | 0.27 | 0.03 |
| JU1409 | 0.20 | 0.07 | 0.09 | 0.06 | 0.17 | 0.05 | 0.10 | 0.02 | 0.08 | 0.02 |
| JU1410 | 0.48 | 0.12 | 0.42 | 0.04 | 0.22 | 0.11 | 0.58 | 0.14 | 0.28 | 0.08 |
| JU1411 | 0.34 | 0.04 | 0.37 | 0.03 | 0.29 | 0.09 | 0.54 | 0.23 | 0.43 | 0.06 |
| JU1416 | 0.54 | 0.06 | 0.39 | 0.05 | 0.11 | 0.08 | 0.53 | 0.06 | 0.07 | 0.03 |
| JU1442 | 0.09 | 0.02 | 0.19 | 0.12 | 0.08 | 0.02 | 0.95 | 0.03 | 0.19 | 0.19 |
| JU1494 | NA | NA | 0.05 | 0.02 | 0.16 | 0.02 | 0.14 | 0.02 | 0.55 | 0.12 |
| JU262 | 0.45 | 0.11 | 0.48 | 0.08 | 0.10 | 0.03 | 0.33 | 0.05 | 0.39 | 0.14 |
| JU319 | 0.04 | 0.04 | 0.03 | 0.01 | 0.09 | 0.05 | 0.53 | 0.21 | 0.00 | 0.00 |
| JU345 | 0.00 | 0.00 | 0.00 | 0.00 | 0.30 | 0.04 | 0.22 | 0.15 | 0.19 | 0.07 |
| JU362 | 0.59 | 0.18 | 0.00 | 0.00 | 0.50 | 0.01 | 0.76 | 0.07 | 0.24 | 0.06 |
| JU393 | 0.45 | 0.28 | 0.03 | 0.01 | 0.36 | 0.07 | 0.87 | 0.07 | 0.20 | 0.05 |
| JU400 | 0.29 | 0.19 | 0.44 | 0.28 | 0.26 | 0.08 | 0.65 | 0.16 | 1.00 | 0.00 |
| MY1 | 0.34 | 0.30 | 0.00 | 0.00 | 0.18 | 0.12 | 0.89 | 0.01 | 0.03 | 0.02 |
| MY16 | 0.10 | 0.06 | 0.28 | 0.06 | 0.15 | 0.03 | 0.27 | 0.19 | 0.99 | 0.01 |
| MY2 | 0.38 | 0.09 | 0.05 | 0.03 | 0.09 | 0.02 | 0.08 | 0.07 | 0.17 | 0.05 |
| PX179 | 0.21 | 0.14 | 0.16 | 0.08 | 0.05 | 0.05 | 0.39 | 0.12 | 0.93 | 0.07 |
| PX174 | 0.07 | 0.06 | 0.07 | 0.02 | 0.09 | 0.02 | 0.54 | 0.16 | 0.26 | 0.11 |

**(b)**

|  | **JU1409** | | **JU1410** | | **MY1** | | **N2** | | **PX174** | |
| --- | --- | --- | --- | --- | --- | --- | --- | --- | --- | --- |
|  | Mean | SE | Mean | SE | Mean | SE | Mean | SE | Mean | SE |
| N2 | 0.01 | 0.01 | 0.11 | 0.03 | 0.08 | 0.04 | 0.28 | 0.05 | 0.01 | 0.01 |
| CB4853 | 0.19 | 0.13 | 0.01 | 0.01 | 0.01 | 0.01 | 0.00 | 0.00 | 0.02 | 0.01 |
| JU1400 | 0.09 | 0.03 | 0.04 | 0.02 | 0.10 | 0.10 | 0.32 | 0.04 | 0.04 | 0.03 |
| JU1401 | 0.18 | 0.03 | 0.15 | 0.06 | 0.48 | 0.01 | 0.21 | 0.12 | 0.41 | 0.01 |
| JU1409 | 0.78 | 0.04 | 0.08 | 0.05 | 0.03 | 0.03 | 0.40 | 0.17 | 0.53 | 0.13 |
| JU1410 | 0.12 | 0.05 | 0.52 | 0.07 | 0.11 | 0.06 | 0.76 | 0.03 | 0.55 | 0.17 |
| JU1411 | 0.10 | 0.07 | 0.20 | 0.04 | 0.05 | 0.02 | 0.53 | 0.04 | 0.51 | 0.02 |
| JU1416 | 0.21 | 0.03 | 0.08 | 0.02 | 0.07 | 0.04 | 0.89 | 0.05 | 0.06 | 0.02 |
| JU1442 | 0.08 | 0.03 | 0.28 | 0.12 | 0.11 | 0.06 | 0.31 | 0.20 | 0.01 | 0.01 |
| JU1494 | NA | NA | 0.03 | 0.01 | 0.28 | 0.11 | 0.32 | 0.08 | 0.01 | 0.01 |
| JU262 | 0.65 | 0.02 | 0.16 | 0.02 | 0.13 | 0.06 | 0.65 | 0.07 | 0.89 | 0.01 |
| JU319 | 0.05 | 0.05 | 0.03 | 0.01 | 0.10 | 0.05 | 0.38 | 0.11 | 0.31 | 0.09 |
| JU345 | 0.03 | 0.02 | 0.02 | 0.02 | 0.35 | 0.02 | 0.31 | 0.25 | 0.10 | 0.06 |
| JU362 | 0.45 | 0.11 | 0.02 | 0.01 | 0.46 | 0.03 | 0.54 | 0.22 | 0.12 | 0.02 |
| JU393 | 0.07 | 0.07 | 0.01 | 0.01 | 0.29 | 0.09 | 0.94 | 0.01 | 0.13 | 0.08 |
| JU400 | 0.00 | 0.00 | 0.34 | 0.05 | 0.26 | 0.07 | 0.73 | 0.12 | 1.00 | 0.00 |
| MY1 | 0.09 | 0.05 | 0.03 | 0.03 | 0.16 | 0.10 | 0.71 | 0.08 | 0.01 | 0.01 |
| MY16 | 0.00 | 0.00 | 0.06 | 0.04 | 0.15 | 0.03 | 0.18 | 0.06 | 1.00 | 0.00 |
| MY2 | 0.05 | 0.05 | 0.01 | 0.01 | 0.06 | 0.04 | 0.20 | 0.12 | 0.19 | 0.16 |
| PX179 | 0.00 | 0.00 | 0.02 | 0.02 | 0.08 | 0.04 | 0.30 | 0.07 | 1.00 | 0.00 |
| PX174 | 0.15 | 0.02 | 0.01 | 0.01 | 0.20 | 0.03 | 0.38 | 0.20 | 0.03 | 0.01 |

**Additional File Table 7.** The 50 randomly selected *C. elegans* genes showing the start and finish positions for each gene identified by its WormBase ID from WS235. To choose these genes, all 20,393 *C. elegans* genes were obtained from ftp://ftp.wormbase.org/pub/wormbase/releases/WS235/species/c_elegans/annotation/c_elegans.WS235.gene_ids.txt.gz and random numbers used for selection

| **Gene ID** | **Public name** | **Linkage group** | **Start (bp)** | **Finish (bp)** |
| --- | --- | --- | --- | --- |
| WBGene00000108 | alh-2 | V | 1644377 | 1647728 |
| WBGene00001468 | flr-4 | X | 15089327 | 15093515 |
| WBGene00002295 | let-19 | II | 8770920 | 8781966 |
| WBGene00003716 | nhr-126 | V | 2229080 | 2230983 |
| WBGene00004235 | ptr-21 | I | 550711 | 556685 |
| [WBGene00005044](http://www.wormbase.org/db/gene/gene?name=WBGene00005044;class=Gene) | sra-18 | I | 12692159 | 12693714 |
| WBGene00005591 | srj-1 | IV | 11963887 | 11965404 |
| WBGene00006104 | str-39 | V | 2877802 | 2879899 |
| WBGene00006440 | tag-63 | I | 3439110 | 3449179 |
| WBGene00006780 | unc-44 | IV | 5966644 | 6004847 |
| WBGene00007058 | dmd-6 | IV | 14616216 | 14623936 |
| WBGene00008577 | F08G2.5 | II | 13832784 | 13833562 |
| WBGene00008962 | F19H6.7 | X | 12377766 | 12378282 |
| WBGene00009422 | F35E8.9 | V | 15916315 | 15917539 |
| WBGene00009858 | thn-4 | V | 16870720 | 16871465 |
| WBGene00010048 | F54D5.2 | II | 11573146 | 11574931 |
| WBGene00010479 | K01G5.8 | III | 10740181 | 10740673 |
| WBGene00010678 | K08F4.3 | IV | 10129253 | 10130225 |
| WBGene00010830 | M02G9.1 | II | 10326070 | 10335702 |
| WBGene00011027 | R05D7.2 | I | 12165720 | 12168163 |
| WBGene00011076 | scav-5 | X | 9858307 | 9861677 |
| WBGene00011676 | cyp-13A2 | II | 9783759 | 9787339 |
| WBGene00011737 | T12G3.1 | IV | 12034230 | 12037029 |
| WBGene00012617 | Y38H6C.4 | V | 20504844 | 20505066 |
| WBGene00013184 | Y53H1B.6 | I | 11303731 | 11305210 |
| WBGene00013570 | Y75B12B.8 | V | 15201457 | 15202367 |
| WBGene00014143 | ZK899.5 | X | 9459852 | 9460877 |
| WBGene00015024 | B0205.10 | I | 10708352 | 10710364 |
| WBGene00015274 | ztf-12 | V | 8786529 | 8792753 |
| WBGene00015400 | cyp-35A2 | V | 7362298 | 7364186 |
| WBGene00015890 | C17C3.5 | II | 5550152 | 5552318 |
| WBGene00016175 | C27H5.6 | II | 7185236 | 7187169 |
| WBGene00017039 | trk-1 | X | 8941341 | 8950924 |
| WBGene00018333 | cyp-33E3 | IV | 8615009 | 8616008 |
| WBGene00019176 | H10D12.2 | IV | 6147528 | 6149916 |
| WBGene00019395 | K04F10.2 | I | 6360846 | 6364062 |
| WBGene00020136 | T01B6.4 | X | 2337648 | 2338298 |
| WBGene00020413 | T10E9.3 | I | 6534073 | 6539192 |
| WBGene00020950 | W02F12.5 | V | 6707037 | 6709086 |
| WBGene00021752 | Y50D7A.2 | III | 268457 | 282881 |
| WBGene00021936 | Y55F3BL.2 | IV | 808674 | 824204 |
| WBGene00022010 | catp-7 | IV | 8585529 | 8594603 |
| WBGene00022709 | ZK354.8 | IV | 5303980 | 5306127 |
| WBGene00043534 | W02B12.13 | II | 11445225 | 11448835 |
| WBGene00044299 | C42D8.9 | X | 5106229 | 5107008 |
| WBGene00044566 | T06F4.3 | X | 4078213 | 4081022 |
| WBGene00045480 | F14F3.5 | X | 10527940 | 10529189 |
| WBGene00194926 | K08D8.12 | IV | 12893552 | 12894700 |
| WBGene00194985 | Y25C1A.14 | II | 3096067 | 3096960 |
| WBGene00195146 | F54D5.17 | II | 11555818 | 11557122 |

**Additional File Table 8.** The genetic distance, as the number of base differences per site between sequences (and SE), among the 20 *C. elegans* lines. All positions containing gaps and missing data were eliminated. There were a total of 183056 positions in the final dataset.

| **Line 1** | **Line 2** | **Distance** | **SE** |
| --- | --- | --- | --- |
| MY16 | JU1401 | 0.00127830 | 0.00004795 |
| MY16 | JU1400 | 0.00185736 | 0.00006387 |
| JU1401 | JU1400 | 0.00172625 | 0.00005825 |
| MY16 | JU319 | 0.00205402 | 0.00006885 |
| JU1401 | JU319 | 0.00104886 | 0.00004450 |
| JU1400 | JU319 | 0.00085220 | 0.00003826 |
| MY16 | JU262 | 0.00160607 | 0.00005885 |
| JU1401 | JU262 | 0.00076479 | 0.00003939 |
| JU1400 | JU262 | 0.00181365 | 0.00005894 |
| JU319 | JU262 | 0.00119089 | 0.00004609 |
| MY16 | JU345 | 0.00225068 | 0.00007266 |
| JU1401 | JU345 | 0.00156236 | 0.00005852 |
| JU1400 | JU345 | 0.00172625 | 0.00006001 |
| JU319 | JU345 | 0.00102701 | 0.00004580 |
| JU262 | JU345 | 0.00134385 | 0.00005793 |
| MY16 | JU362 | 0.00129469 | 0.00005007 |
| JU1401 | JU362 | 0.00001639 | 0.00000722 |
| JU1400 | JU362 | 0.00174264 | 0.00005705 |
| JU319 | JU362 | 0.00106525 | 0.00004570 |
| JU262 | JU362 | 0.00078118 | 0.00004239 |
| JU345 | JU362 | 0.00157875 | 0.00005917 |
| MY16 | JU393 | 0.00117997 | 0.00004359 |
| JU1401 | JU393 | 0.00051350 | 0.00003314 |
| JU1400 | JU393 | 0.00179180 | 0.00006970 |
| JU319 | JU393 | 0.00109256 | 0.00005452 |
| JU262 | JU393 | 0.00063369 | 0.00003282 |
| JU345 | JU393 | 0.00150774 | 0.00004763 |
| JU362 | JU393 | 0.00052989 | 0.00003259 |
| MY16 | JU400 | 0.00184643 | 0.00006187 |
| JU1401 | JU400 | 0.00171532 | 0.00006152 |
| JU1400 | JU400 | 0.00002185 | 0.00000623 |
| JU319 | JU400 | 0.00085220 | 0.00003871 |
| JU262 | JU400 | 0.00180273 | 0.00006141 |
| JU345 | JU400 | 0.00171532 | 0.00005970 |
| JU362 | JU400 | 0.00173171 | 0.00006017 |
| JU393 | JU400 | 0.00178088 | 0.00007349 |
| MY16 | JU1409 | 0.00127830 | 0.00004795 |
| JU1401 | JU1409 | 0.00000000 | 0.00000000 |
| JU1400 | JU1409 | 0.00172625 | 0.00005825 |
| JU319 | JU1409 | 0.00104886 | 0.00004450 |
| JU262 | JU1409 | 0.00076479 | 0.00003939 |
| JU345 | JU1409 | 0.00156236 | 0.00005852 |
| JU362 | JU1409 | 0.00001639 | 0.00000722 |
| JU393 | JU1409 | 0.00051350 | 0.00003314 |
| JU400 | JU1409 | 0.00171532 | 0.00006152 |
| MY16 | JU1410 | 0.00131107 | 0.00004980 |
| JU1401 | JU1410 | 0.00005463 | 0.00001098 |
| JU1400 | JU1410 | 0.00178088 | 0.00005993 |
| JU319 | JU1410 | 0.00110349 | 0.00004640 |
| JU262 | JU1410 | 0.00081942 | 0.00003983 |
| JU345 | JU1410 | 0.00161699 | 0.00005942 |
| JU362 | JU1410 | 0.00007102 | 0.00001259 |
| JU393 | JU1410 | 0.00056813 | 0.00003637 |
| JU400 | JU1410 | 0.00176995 | 0.00006330 |
| JU1409 | JU1410 | 0.00005463 | 0.00001098 |
| MY16 | JU1411 | 0.00127830 | 0.00004795 |
| JU1401 | JU1411 | 0.00000000 | 0.00000000 |
| JU1400 | JU1411 | 0.00172625 | 0.00005825 |
| JU319 | JU1411 | 0.00104886 | 0.00004450 |
| JU262 | JU1411 | 0.00076479 | 0.00003939 |
| JU345 | JU1411 | 0.00156236 | 0.00005852 |
| JU362 | JU1411 | 0.00001639 | 0.00000722 |
| JU393 | JU1411 | 0.00051350 | 0.00003314 |
| JU400 | JU1411 | 0.00171532 | 0.00006152 |
| JU1409 | JU1411 | 0.00000000 | 0.00000000 |
| JU1410 | JU1411 | 0.00005463 | 0.00001098 |
| MY16 | JU1416 | 0.00127830 | 0.00004795 |
| JU1401 | JU1416 | 0.00000000 | 0.00000000 |
| JU1400 | JU1416 | 0.00172625 | 0.00005825 |
| JU319 | JU1416 | 0.00104886 | 0.00004450 |
| JU262 | JU1416 | 0.00076479 | 0.00003939 |
| JU345 | JU1416 | 0.00156236 | 0.00005852 |
| JU362 | JU1416 | 0.00001639 | 0.00000722 |
| JU393 | JU1416 | 0.00051350 | 0.00003314 |
| JU400 | JU1416 | 0.00171532 | 0.00006152 |
| JU1409 | JU1416 | 0.00000000 | 0.00000000 |
| JU1410 | JU1416 | 0.00005463 | 0.00001098 |
| JU1411 | JU1416 | 0.00000000 | 0.00000000 |
| MY16 | JU1442 | 0.00114719 | 0.00004815 |
| JU1401 | JU1442 | 0.00044795 | 0.00003408 |
| JU1400 | JU1442 | 0.00180273 | 0.00006783 |
| JU319 | JU1442 | 0.00108164 | 0.00005532 |
| JU262 | JU1442 | 0.00067739 | 0.00003454 |
| JU345 | JU1442 | 0.00151866 | 0.00005103 |
| JU362 | JU1442 | 0.00046434 | 0.00003765 |
| JU393 | JU1442 | 0.00027314 | 0.00002210 |
| JU400 | JU1442 | 0.00179180 | 0.00007130 |
| JU1409 | JU1442 | 0.00044795 | 0.00003408 |
| JU1410 | JU1442 | 0.00048073 | 0.00003668 |
| JU1411 | JU1442 | 0.00044795 | 0.00003408 |
| JU1416 | JU1442 | 0.00044795 | 0.00003408 |
| MY16 | JU1494 | 0.00144764 | 0.00004950 |
| JU1401 | JU1494 | 0.00062822 | 0.00003615 |
| JU1400 | JU1494 | 0.00200485 | 0.00007065 |
| JU319 | JU1494 | 0.00133839 | 0.00005175 |
| JU262 | JU1494 | 0.00072109 | 0.00003356 |
| JU345 | JU1494 | 0.00154051 | 0.00004994 |
| JU362 | JU1494 | 0.00064461 | 0.00003627 |
| JU393 | JU1494 | 0.00042064 | 0.00003640 |
| JU400 | JU1494 | 0.00199393 | 0.00007419 |
| JU1409 | JU1494 | 0.00062822 | 0.00003615 |
| JU1410 | JU1494 | 0.00068285 | 0.00003714 |
| JU1411 | JU1494 | 0.00062822 | 0.00003615 |
| JU1416 | JU1494 | 0.00062822 | 0.00003615 |
| JU1442 | JU1494 | 0.00045341 | 0.00003526 |
| MY16 | MY1 | 0.00134931 | 0.00005445 |
| JU1401 | MY1 | 0.00061730 | 0.00003297 |
| JU1400 | MY1 | 0.00174264 | 0.00006112 |
| JU319 | MY1 | 0.00134931 | 0.00005343 |
| JU262 | MY1 | 0.00085766 | 0.00004268 |
| JU345 | MY1 | 0.00178634 | 0.00006378 |
| JU362 | MY1 | 0.00063369 | 0.00003300 |
| JU393 | MY1 | 0.00073748 | 0.00004127 |
| JU400 | MY1 | 0.00173171 | 0.00006437 |
| JU1409 | MY1 | 0.00061730 | 0.00003297 |
| JU1410 | MY1 | 0.00065007 | 0.00003333 |
| JU1411 | MY1 | 0.00061730 | 0.00003297 |
| JU1416 | MY1 | 0.00061730 | 0.00003297 |
| JU1442 | MY1 | 0.00061730 | 0.00003508 |
| JU1494 | MY1 | 0.00091775 | 0.00004296 |
| MY16 | MY2 | 0.00136024 | 0.00005442 |
| JU1401 | MY2 | 0.00062822 | 0.00003312 |
| JU1400 | MY2 | 0.00175356 | 0.00005940 |
| JU319 | MY2 | 0.00136024 | 0.00005200 |
| JU262 | MY2 | 0.00086859 | 0.00004378 |
| JU345 | MY2 | 0.00179726 | 0.00006220 |
| JU362 | MY2 | 0.00064461 | 0.00003319 |
| JU393 | MY2 | 0.00074840 | 0.00004165 |
| JU400 | MY2 | 0.00174264 | 0.00006241 |
| JU1409 | MY2 | 0.00062822 | 0.00003312 |
| JU1410 | MY2 | 0.00066100 | 0.00003331 |
| JU1411 | MY2 | 0.00062822 | 0.00003312 |
| JU1416 | MY2 | 0.00062822 | 0.00003312 |
| JU1442 | MY2 | 0.00062822 | 0.00003549 |
| JU1494 | MY2 | 0.00092868 | 0.00004418 |
| MY1 | MY2 | 0.00002731 | 0.00000635 |
| MY16 | N2 | 0.00105432 | 0.00004921 |
| JU1401 | N2 | 0.00044249 | 0.00002905 |
| JU1400 | N2 | 0.00192837 | 0.00006212 |
| JU319 | N2 | 0.00121821 | 0.00005611 |
| JU262 | N2 | 0.00081396 | 0.00004529 |
| JU345 | N2 | 0.00127283 | 0.00004929 |
| JU362 | N2 | 0.00045888 | 0.00003229 |
| JU393 | N2 | 0.00040971 | 0.00002953 |
| JU400 | N2 | 0.00191745 | 0.00006469 |
| JU1409 | N2 | 0.00044249 | 0.00002905 |
| JU1410 | N2 | 0.00047526 | 0.00003195 |
| JU1411 | N2 | 0.00044249 | 0.00002905 |
| JU1416 | N2 | 0.00044249 | 0.00002905 |
| JU1442 | N2 | 0.00025675 | 0.00002716 |
| JU1494 | N2 | 0.00061183 | 0.00003844 |
| MY1 | N2 | 0.00052443 | 0.00003693 |
| MY2 | N2 | 0.00053536 | 0.00003685 |
| MY16 | PX174 | 0.00178634 | 0.00005878 |
| JU1401 | PX174 | 0.00116358 | 0.00004270 |
| JU1400 | PX174 | 0.00264946 | 0.00007559 |
| JU319 | PX174 | 0.00197207 | 0.00006859 |
| JU262 | PX174 | 0.00156783 | 0.00005569 |
| JU345 | PX174 | 0.00216874 | 0.00007181 |
| JU362 | PX174 | 0.00117997 | 0.00004419 |
| JU393 | PX174 | 0.00120728 | 0.00004272 |
| JU400 | PX174 | 0.00263854 | 0.00007830 |
| JU1409 | PX174 | 0.00116358 | 0.00004270 |
| JU1410 | PX174 | 0.00118543 | 0.00004556 |
| JU1411 | PX174 | 0.00116358 | 0.00004270 |
| JU1416 | PX174 | 0.00116358 | 0.00004270 |
| JU1442 | PX174 | 0.00097784 | 0.00004061 |
| JU1494 | PX174 | 0.00132200 | 0.00005001 |
| MY1 | PX174 | 0.00126737 | 0.00004652 |
| MY2 | PX174 | 0.00127830 | 0.00004560 |
| N2 | PX174 | 0.00090683 | 0.00004076 |
| MY16 | PX179 | 0.00108710 | 0.00005048 |
| JU1401 | PX179 | 0.00037693 | 0.00002911 |
| JU1400 | PX179 | 0.00193930 | 0.00006439 |
| JU319 | PX179 | 0.00122913 | 0.00005922 |
| JU262 | PX179 | 0.00082488 | 0.00004475 |
| JU345 | PX179 | 0.00136024 | 0.00005207 |
| JU362 | PX179 | 0.00038240 | 0.00003068 |
| JU393 | PX179 | 0.00044249 | 0.00002641 |
| JU400 | PX179 | 0.00192837 | 0.00006715 |
| JU1409 | PX179 | 0.00037693 | 0.00002911 |
| JU1410 | PX179 | 0.00040971 | 0.00003166 |
| JU1411 | PX179 | 0.00037693 | 0.00002911 |
| JU1416 | PX179 | 0.00037693 | 0.00002911 |
| JU1442 | PX179 | 0.00021305 | 0.00002412 |
| JU1494 | PX179 | 0.00056813 | 0.00003565 |
| MY1 | PX179 | 0.00053536 | 0.00003385 |
| MY2 | PX179 | 0.00054628 | 0.00003344 |
| N2 | PX179 | 0.00009833 | 0.00001200 |
| PX174 | PX179 | 0.00089590 | 0.00004143 |

**Additional Files section 2. Ascaroside synthesis**

Solvents used were commercial grade and dried via passage through a Grubbs type system (Anhydrous engineering). The removal of solvents *in vacuo* was achieved using a Büchi rotary evaporator (bath temperatures up to 40 °C) at a pressure of 15 mmHg (diaphragm pump) or at 6 mmHg (oil pump) on a vacuum line. Reagents were commercial grade and used as received. Deuterated solvents for NMR analysis were purchased from Cambridge Isotopes Limited*.* NMR spectra were recorded on Varian 400, Varian 500, JEOL GX 300 or Eclipse 300 spectrometers. All chemical shifts were quoted in parts per million (ppm); ^1^H and ^13^C NMR spectra were referenced to TMS as an internal standard. The following abbreviations (and their combinations) were used to label the multiplicities: s (singlet), d (doublet), t (triplet), m (multiplet). Coupling constants, *J*, in the ^1^H NMR spectra were calculated using ACDLabs*,* and are reported to the nearest 0.1 Hz. Assignment of signals in ^1^H and ^13^C NMR were performed using ^1^H-^1^H COSY, HMQC, HSQC, HMBC and TOCSY experiments, where appropriate. Mass spectra for characterisation were acquired by the University of Bristol mass spectrometry service using electrospray ionisation (ESI+/-) using a Bruker Daltonics Apex IV spectrometer. The synthesis scheme is shown in the Figure.

**Figure.** Reagents and conditions. i) BzCl, THF, DMAP, Et_3_N, 98%; ii) NH_3_, MeOH, THF, RT, 53%; iii) [PyrH][CrCl_3_O], CH_2_Cl_2_, 84%; iv) DBU, DCM, -78 °C, 89%; v) Pd/C, H_2_, EtOAc, 80%; vi) disiamylborane, THF, 0°C, 81%; vii) Cl_3_CCN, DBU, CH_2_Cl_2_, 80%; viii) 2*R*,5*R*-hexan-2,5-diol, TMSOTf, CH_2_Cl_2_, 78%; ix) 1M NaOH, MeOH, then NaHCO_3_, 85%; 2*R*-oct-7-en-2-ol, x) TMSOTf, CH_2_Cl_2_, 82%; 3 mol% Grubbs II, CH_2_Cl_2_; xi) 1M NaOH, MeOH; xii) 2M HCl, Et_2_O, 0 °C, 75% (steps x-xii).

For ascr#3, alkenyl ether **2** (0.531g, 1.14 mmol) was dissolved in dry DCM under an N_2_ atmosphere and then acrylic acid (0.39 mL, 5.70 mmol) was added, followed by Grubbs second-generation ruthenium metathesis catalyst (29 mg, 0.034 mmol). The reaction mixture was heated under reflux overnight, then cooled to ambient temperature and the volatiles removed under vacuum. Purified by column chromatography on silica-gel eluting with a 95/5 v/v mixture of dichloromethane and ethanol gave the dibenzoate of ascr#3 as a brown oil (625 mg; yield >100%, contaminated with residual acrylic acid). ^1^H NMR (400 MHz, CDCl_3_): 8.10 (m, 2 H); 8.04 (m, 2 H); 7.57 (m, 2H); 7.46 (m, 4 H); 7.10 (dt, 15.6 Hz, 6.8 Hz, 1 H); 5.85 (d, 15.6 Hz, 1 H); 5.16 (m, 1 H); 4.96 (s, 1 H); 4.1 (m, 1 H); 3.85 (m, 1 H); 2.42 (dt, 13.1 Hz, 3.8 Hz, 1 H); 2.29 (m, 1 H); 2.20 (m, 1 H); 1.66 (m, 1 H); 1.54 (m, 5 H); 1.29 (d, 6.0 Hz, 3 H); 1.20 (d, 6.4 Hz, 3H). ^13^C NMR (100 MHz, CDCl_3_): 171.4; 165.6; 165.5; 151.9; 133.2; 133.1; 129.8; 129.6; 128.4; 120.7; 93.8; 72.5; 71.2; 70.6; 67.0; 36.8; 32.2; 29.2; 22.8; 25.27; 19.1; 17.9. A cold solution of 1M NaOH in methanol (15 mL) was added to the dibenzoate (592 mg, 1.16 mmol) under N_2_ and the reaction mixture was stirred at 0 °C for 2h. Then, 5 mL of a 2M solution of anhydrous HCl in Et_2_O was added. The volatiles were removed under vacuum and the residue purified by column chromatography on silica-gel eluting with a 4/1 v/v mixture of dichloromethane and ethanol to give pheromone ascr#3 as a brown oil (259 mg, 75%). ^1^H NMR (400 MHz, D_2_O): 6.95 (dt, 15.6 Hz, 6.8 Hz, 1 H); 5.73 (d, 15.6 Hz, 1 H); 4.54 (s, 1 H); 3.70 (m, 1 H); 3.62 (m, 1 H); 3.5 (m, 1 H); 3.42 (ddd, 4.4 Hz, 10.8 Hz, 11.2 Hz, 1H); 2.14 (m, 1 H); 1.85 (dt, 13.2 Hz, 4 Hz, 1 H); 1.66 (ddd, 2.8 Hz, 11.2 Hz, 13.2 Hz, 1 H); 1.40 (m, 6 H); 1.12 (d, 6.4 Hz, 3 H); 1.02 (d, 6 Hz, 3H). ^13^C NMR (100 MHz, DMSO): 141.6; 88.0; 62.9; 61.2; 60.4: 58.7; 28.4; 26.4; 23.6; 19.6; 16.9; 9.8; 8.6. ESI m/z = 325.2 [M+Na]^+^.

**Additional Files section 3. Quantification of ascr#2 and ascr#3 in natural pheromone mixtures.**

After sequential dilution (see below) the resulting solutions were analysed by HPLC-MS as follows. Samples (20 mL in methanol) were injected onto a Waters 2795HT HPLC system. Detection was achieved by uv between 200 and 410 nm using a Waters 996 diode array detector, and by simultaneous electrospray (ES^+^) mass spectrometry using a Waters ZQ spectrometer in SIR mode for the following ions: *m/z* 375; *m/z* 325; and *m/z* 269. Chromatography (flow rate 1 mL·min^-1^) was achieved using a Phenomenex Kinetex column (2.6 μ, C_18_, 100 Å, 4.6 × 100 mm) equipped with a Phenomenex Security Guard precolumn (Luna C_5_ 300 Å). The base solvent mix was HPLC grade H_2_O containing 0.05% formic acid. The gradient was established by co-mixing HPLC grade CH_3_CN containing 0.045% formic acid in varying proportions. The gradient was: 0 min, 90% base, 10% co-mix; 10 min, 10% base, 90% co-mix; 12 min, 10% base, 90% co-mix; 13min, 90% base, 10% co-mix; 15 min, 90% base, 10% co-mix.

Calibration curves (Figure, below) were generated using pure synthetic samples of **S** (*m/z* [M+Na] 375, retention time 10.15 minutes), ascr#3 (*m/z* [M+Na] 325, retention time 4.33 minutes), and ascr#2 (*m/z* [M+Na] 269, retention time 2.85 minutes) to establish absolute and relative response factors (as ion intensity at the appropriate SIR channel m/z) and to establish the zones of linearity of concentration versus intensity. The pheromone extracts were analysed, then diluted by a factor of two and reanalysed, this process being repeated until signal to noise was unsatisfactory in the resulting m/z intensity data collected by HPLC-MS. From these data the ratios of ascr#2 to ascr#3 were established, using at least three of the dilution sets within the linear zones. Separate weighed samples of the dried natural pheromone mixture were then suspended in methanol, an accurate volume of a known concentration of stock solution of the standard (**S**) in methanol was added and the mixtures analysed by HPLC-MS. Using the response factor relative to **S**, the data were processed to deduce the mmoles of ascr#3 and ascr#2 in the original total volume of culture and then normalised as the quantity of pheromones ascr#3 and ascr#2 produced per worm (mmoles/worm).

**Figure**. Calibration curves for LC-MS analysis of pheromones ascr#3 and ascr#2 versus standard **S**. *x*-axis: concentration of sample in reference stock solutions of pure materials in methanol; *y*-axis ion-intensity from chromatograms with single ion-monitoring at *m/z* 375 (**S**); *m/z* 325 (ascr#3); and *m/z* 269 (ascr#2); all three species as Na^+^ adducts. The concentration (mM) is that in the 20 mL injection samples (in methanol).

**Additional Files section 4. Sequence Information**

The sequence information for each *C. elegans* line for the genes shown in Additional File section 1 Table 7 is in file nebc.nerc.ac.uk:nebcfs:Viney/Additional_Files_section_4.txt available at http://nebc.nerc.ac.uk/nebcfs/public/Viney/Additional_Files_section_4.txt
